# JAM-A interacts with α3β1 integrin and tetraspanins CD151 and CD9 to regulate collective cell migration of polarized epithelial cells

**DOI:** 10.1007/s00018-022-04140-5

**Published:** 2022-01-24

**Authors:** Sonja Thölmann, Jochen Seebach, Tetsuhisa Otani, Luise Florin, Hans Schnittler, Volker Gerke, Mikio Furuse, Klaus Ebnet

**Affiliations:** 1 Institute-Associated Research Group “Cell Adhesion and Cell Polarity”, Institute of Medical Biochemistry, ZMBE, University of Münster, Von-Esmarch-Str. 56, 48149 Münster, Germany; 2 Institute of Medical Biochemistry, ZMBE, University of Münster, Münster, Germany; 3 Institute of Anatomy and Vascular Biology, University of Münster, Münster, Germany; 4 Cells-in-Motion Interfaculty Center, University of Münster, 48149 Münster, Germany; 5 Division of Cell Structure, National Institute for Physiological Sciences, National Institute of Natural Sciences, Okazaki, Aichi Japan; 6 Institute for Virology and Research Center for Immunotherapy (FZI), University Medical Center of the Johannes Gutenberg-University Mainz, Mainz, Germany

**Keywords:** Cell polarity, Cryptic lamellipodia, Junctional adhesion molecules, JAMs, MDCK, Tetraspanins

## Abstract

Junctional adhesion molecule (JAM)-A is a cell adhesion receptor localized at epithelial cell–cell contacts with enrichment at the tight junctions. Its role during cell–cell contact formation and epithelial barrier formation has intensively been studied. In contrast, its role during collective cell migration is largely unexplored. Here, we show that JAM-A regulates collective cell migration of polarized epithelial cells. Depletion of JAM-A in MDCK cells enhances the motility of singly migrating cells but reduces cell motility of cells embedded in a collective by impairing the dynamics of cryptic lamellipodia formation. This activity of JAM-A is observed in cells grown on laminin and collagen-I but not on fibronectin or vitronectin. Accordingly, we find that JAM-A exists in a complex with the laminin- and collagen-I-binding α3β1 integrin. We also find that JAM-A interacts with tetraspanins CD151 and CD9, which both interact with α3β1 integrin and regulate α3β1 integrin activity in different contexts. Mapping experiments indicate that JAM-A associates with α3β1 integrin and tetraspanins CD151 and CD9 through its extracellular domain. Similar to depletion of JAM-A, depletion of either α3β1 integrin or tetraspanins CD151 and CD9 in MDCK cells slows down collective cell migration. Our findings suggest that JAM-A exists with α3β1 integrin and tetraspanins CD151 and CD9 in a functional complex to regulate collective cell migration of polarized epithelial cells.

**Supplementary Information:**

The online version contains supplementary material available at 10.1007/s00018-022-04140-5.

## Introduction

Collective cell migration is a process in which groups of cells migrate in a coordinated manner. Movements of cell collectives occur during development, repair and regeneration processes but also during pathophysiological processes like cancer invasion [1, 2]. In contrast to collectively migrating cancer cells, which adopt mesenchymal characteristics and which form loose and rather transient cell–cell junctions, collectively migrating epithelial cells maintain their apico-basal polarity and remain tightly connected through stable intercellular junctions [3].

The coordination of directed migration is enabled by the formation of polarized protrusions beneath the cell migrating in front of the cell, so-called cryptic lamellipodia [4, 5], while at the same time maintaining regular cell–cell junctions at the apical cell–cell junctions. The formation of oriented cryptic lamellipodia requires intact adherens junctions which act as scaffolds for the Arp2/3 complex and its regulator Wave [6]. A structure similar to cryptic lamellipodia has been observed in collectively migrating endothelial cells [7–9]. Similar to cryptic lamellipodia of migrating epithelial cells, their formation requires an intact cadherin/catenin complex indicating an active role of intercellular junctions in the formation of polarized protrusions during collective cell migration.

Recent evidence strongly suggests a role for intercellular junctions in mechanosensing and transduction. Leader cells at the forefront of a cellular collective respond to chemical cues by front-to-rear polarization, i.e., increased protrusive activity at their front and increased actomyosin-driven contractility at their rear end [2]. The forces generated by migrating leader cells are transmitted to the cells behind the front through intercellular junctions-mediated force-sensing and force-transducing mechanisms [10–15]. Molecular components localized at all structures at cell–cell junctions including tight junctions (TJs), adherens junctions (AJs), desmosomes and gap junctions contribute to the behavior of collectives of migrating cells [16]. Interestingly, physical properties associated with forces such as strain energy or monolayer tension and those associated with kinematic behavior such as monolayer velocity are differently influenced by adhesion molecules and their associated cytoplasmic proteins [16], indicating that signaling inputs derived from multiple cell–cell adhesion receptor systems are integrated into coordinated cell behavior during collective cell migration.

Junctional adhesion molecule A (JAM-A) is a member of the immunoglobulin superfamily (IgSF) with a variety of functions in different cells types [17]. In polarized epithelial cells, JAM-A is localized at intercellular junctions and is enriched at TJs where it is phosphorylated by the Par3–aPKC–Par6 polarity protein complex [18]. Depletion of JAM-A in SK-CO15 epithelial cells results in reduced cell migration in scratch-wounding assays [19]. In different tumor cell lines, the levels of JAM-A were found to correlate both positively and negatively with cell migration [20–23] suggesting a context-dependent function of JAM-A during migration. In endothelial cells, JAM-A regulates cell migration in response to bFGF through its interaction with tetraspanin (Tspan) CD9 and αvβ3 integrin [24–26].

In this study, we addressed the role of JAM-A during collective cell migration of polarized epithelial cells. Using MDCKII cells in a monolayer expansion model [16], we find that JAM-A is required for efficient migration of MDCKII cell collectives. Depletion of JAM-A increases the migration velocity of singly migrating MDCKII cells but slows down the migration velocity of MDCKII cell collectives. We find that JAM-A interacts with α3β1 integrin as well as with Tspans CD151 and CD9, two α3β1 integrin binding partners and regulators of α3β1 integrin functions. Our findings suggest a tetraspanin-enriched microdomain (TEM) in which CD151 and CD9 link JAM-A to α3β1 integrin, and identify JAM-A as a regulator of collective cell migration in polarized epithelial cells.

## Results

### JAM-A limits single cell migration in a cell-autonomous manner in polarized epithelial cells

**Fig. 1:**
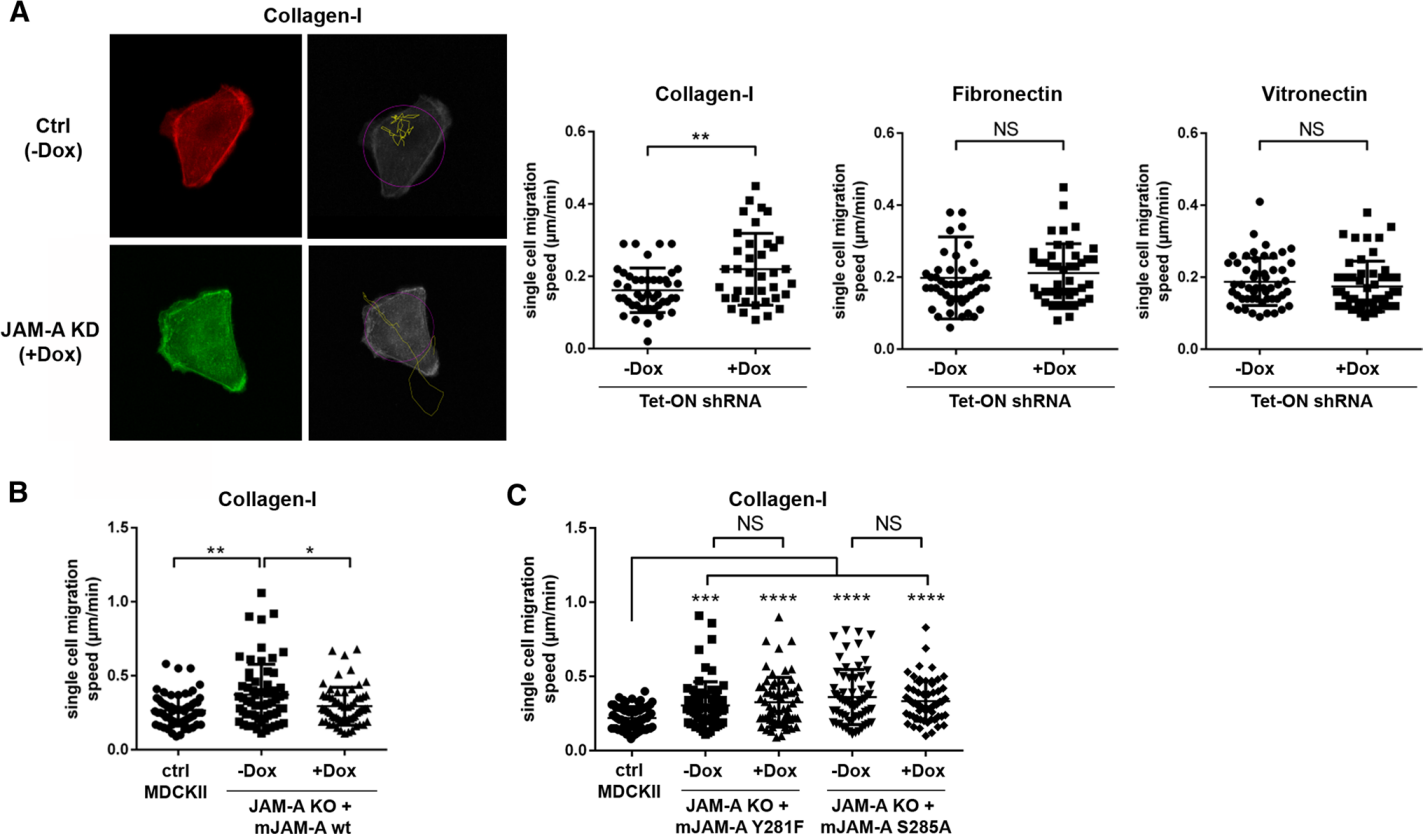
JAM-A limits single cell migration. **A** Single cell migration of MDCKII cells with inducible expression of JAM-A shRNAs (pEmU6proT, −Dox: shRNA not induced, +Dox: shRNA induced). Control cells (−Dox) were stably transfected with LA-mCherry, JAM-A KD cells (+Dox) were stably transfected with LA-EGFP. Cells were cultured on collagen-I (Col-I), fibronectin (FN), or vitronectin (VN) as indicated. Left panels: representative images of a LA-mCherry-transfected control cell (Ctrl, −Dox) and a LA-GFP-transfected JAM-A knockdown cell (JAM-A KD, +Dox) grown on collagen-I. Black-and-white images show the motility paths (yellow lines); the blob diameters used to track the cell during migration are highlighted by purple circles. Quantifications of single cell migration speed are shown in the right panels. Number of cells analyzed: Col-I: −Dox: 45, +Dox: 38, FN: −Dox: 48, +Dox: 44; VN: −Dox: 52, +Dox: 52, five independent experiments. **B** Quantifications of single cell migration speed of JAM-A KO MDCKII cells reconstituted with an inducible expression vector for murine JAM-A (pInducer21, −Dox: not induced, +Dox: induced). As control cells, MDCKII cells with inducible JAM-A shRNA expression in the absence of Dox were used. Number of cells analyzed: Ctrl MDCKII: 63; JAM-A KO, −Dox: 64; JAM-A KO, +Dox: 63; three independent experiments. **C** Quantifications of single cell migration speed of JAM-A KO MDCKII cells reconstituted with an inducible expression vector encoding phospho-deficient mJAM-A mutants (mJAM-A Y281F or mJAM-A S285A in pInducer21, −Dox: not induced, +Dox: induced). Number of cells analyzed: Ctrl MDCKII: 70; JAM-A KO + mJAM-A Y281F: −Dox: 67; +Dox: 65; JAM-A KO + mJAM-A S285A: −Dox: 62; +Dox: 65; four independent experiments). All statistical analyses shown in this figure were performed using Mann–Whitney *U* test. Data are presented as mean values ± SD. *NS* not significant; *P < 0.05, **P < 0.01, ***P < 0.001, ****P < 0.0001

We first analyzed single cell migration of MDCKII cells after depletion of JAM-A using a doxycycline (dox)-regulatable JAM-A shRNA expression system [27] (Suppl. Figure 1). To address a possible contribution of the extracellular matrix (ECM), cells were plated on different ECM components including collagen type I (Col-I), fibronectin (FN) and vitronectin (VN). Cell motility was analyzed by live cell microscopy over a period of 10 h. Surprisingly, JAM-A depletion resulted in a significant increase in migratory speed (Fig. 1A, Suppl. Movie 1, 2) suggesting that JAM-A limits motility at the single cell level. The increase in migration speed after JAM-A depletion was only observed in cells plated on Col-I but not in cells plated on FN or VN suggesting that its function in limiting migratory activity in single cells depends on its functional interaction with a Col-I-binding integrin. To further confirm the increased motility of MDCKII cells in the absence of JAM-A we used JAM-A knockout (KO) MDCKII cells with a CRISPR/Cas9-mediated inactivation of the JAM-A gene [28] (Suppl. Figure 1). As observed after shRNA-mediated downregulation of JAM-A expression, JAM-A gene inactivation resulted in a significantly increased migration velocity which was restored to levels observed in control MDCKII cells by ectopic expression of murine JAM-A (mJAM-A) (Fig. 1B). Interestingly, ectopic expression of mJAM-A constructs with mutations at phosphorylation sites that regulate JAM-A function, i.e., Tyr281 and Ser285 [18, 29–31], did not restore the migratory activity to levels observed in MDCKII WT cells (Fig. 1C). Together, these findings indicated that JAM-A limits the migratory activity in polarized epithelial cells in the absence of cell–cell adhesion, suggesting a cell-autonomous function of JAM-A in cell migration. They also suggested that this function depends on a functional interaction of JAM-A with a Col-I-binding integrin and involves phosphorylation of JAM-A at Tyr281 and at Ser285.

### JAM-A positively regulates collective cell migration in polarized epithelial cells

**Fig. 2:**
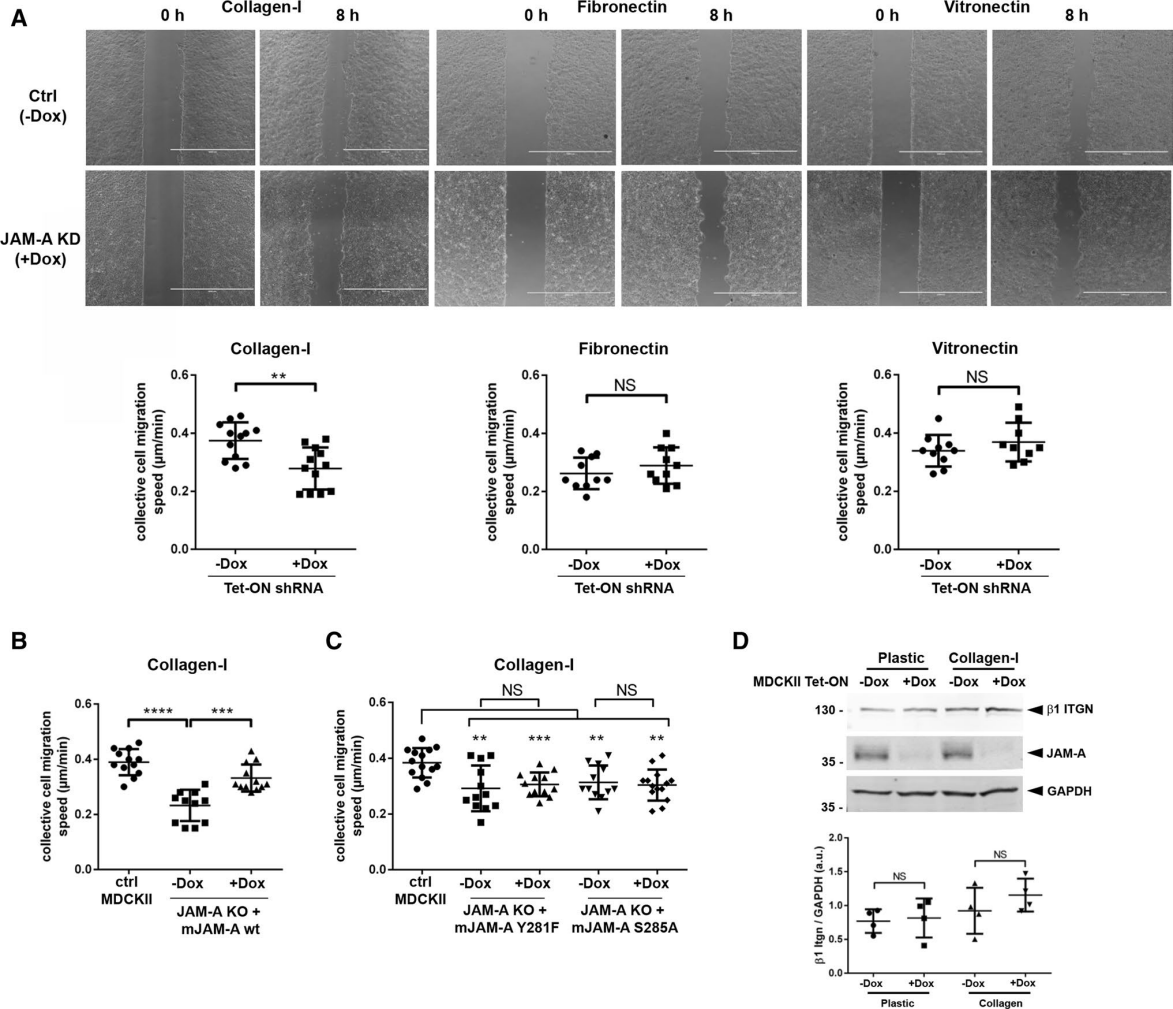
JAM-A positively regulates collective cell migration. **A** Monolayer expansion assays of MDCKII cells with inducible expression of JAM-A shRNAs (described in the legend to Fig. 1). Cells were seeded on Col-I, FN- or VN-coated microscope slides separated by a silicone stamp. Collective migration was triggered by stamp removal. The migration velocity of the cell collective was quantified by measuring the cell-free area directly after the removal of the stamp and 8 h later (see “Materials and Methods” for details). Top panels: representative images of monolayer expansion immediately after stamp removal (0 h) and after 8 h (8 h). Bottom panels: quantifications of collective cell migration velocities on different ECM substrates. Each dot represents one biological replicate (one independent cell population). Number of independent cell populations analyzed: Col-I: *n* = 12 for each cell line (four independent experiments), FN: *n* = 10 for each cell line (three independent experiments), VN: *n* = 9 for each cell line (three independent experiments). **B** Quantifications of collective cell migration speed of JAM-A KO MDCKII cells reconstituted with an inducible expression vector for murine JAM-A (see legend to Fig. 1B). Number of independent cell populations analyzed: *n* = 12 for each condition, three independent experiments. **C** Quantifications of collective cell migration speed of JAM-A KO MDCKII cells reconstituted with an inducible expression vector encoding phospho-deficient mJAM-A mutants (see legend to Fig. 1C). Number of independent cell populations analyzed: *n* = 14 for ctrl MDCKII cells, *n* = 12 for JAM-A KO MDCKII cells and mJAM-A expressing JAM-A KO MDCKII cells; three independent experiments. **D** β1 integrin expression in JAM-A KO MDCKII cells. Top: western blot analysis of β1 integrin in MDCKII cells with doxycycline-regulated expression of JAM-A shRNAs (pEmU6proT, −Dox: shRNA not induced, +Dox: shRNA induced). Cells were grown either on plastic or on collagen-I. Bottom: quantitative analysis of β1 integrin protein levels. Data are presented as mean values ± SD (*n* = 4 independent experiments). All statistical analyses shown in this figure were performed using two-tailed Student’s t test. Data are presented as mean values ± SD. *NS* not significant; **P < 0.01, ***P < 0.001, ****P < 0.0001

We next analyzed the role of JAM-A in collectively migrating cells in a monolayer expansion model [13, 32]. To this, cells were grown to confluency in two-well slides in which individual chambers are separated. After reaching confluency, cell sheet migration was induced by removing the inserts. Migration of the monolayer was monitored over a period of 8 h. In contrast to the observations in single cells, depletion of JAM-A resulted in a reduced migration velocity of the cellular collective (Fig. 2A). This effect was only observed when cells migrated on Col-I but not when cells migrated on FN or on LN (Fig. 2A). The reduction in migration velocity of cell collectives was also observed after CRISPR/Cas9-mediated inactivation of the JAM-A gene (Fig. 2B). Ectopic expression of mJAM-A in these cells almost completely restored the migration velocity to levels observed in control MDCKII cells (Fig. 2B), whereas ectopic expression of mJAM-A/Y281F or mJAM-A/S285A did not restore this function (Fig. 2C). Since collective cell migration depends on functional adherens junctions (AJs) [33], we stained JAM-A KO MDCKII cells with antibodies against various AJ components. We observed no difference between control MDCKII cells and JAM-A KO MDCKII cells in the localization of α-catenin, β-catenin, p120^ctn^, and E-cadherin (Suppl. Figure 2), strongly suggesting that the reduced migration velocity of cell collectives after depletion of JAM-A is not due to an indirect effect of JAM-A depletion on AJ integrity. To test the possibility that JAM-A regulates collective cell migration by influencing focal adhesion dynamics, we analyzed the expression and localization of paxillin, a scaffolding protein at focal adhesions that regulates focal adhesion turnover, cell motility and cell migration [34]. We found that both paxillin protein levels and paxillin-positive focal adhesions were unchanged after deletion of JAM-A (Suppl. Figure 3A, B), suggesting that focal adhesions are not affected by JAM-A depletion. Previous studies indicated that JAM-A regulates collective cell migration in MCF7 breast cancer cells by regulating β1 integrin protein expression [35, 36]. To test if a similar mechanism operates in MDCKII cells, we analyzed the levels of the β1 integrin chain by Western blotting. We observed no difference in β1 integrin levels between control cells and JAM-A KD MDCKII cells (Fig. 2D), suggesting a different molecular mechanism through which JAM-A regulates collective cell migration in polarized epithelial cells. Together, these observations indicated that in contrast to its inhibitory function on cell migration in single cells, JAM-A promotes cell migration when cells migrate as a collective.

### JAM-A regulates cryptic lamellipodia dynamics during collective cell migration

**Fig. 3:**
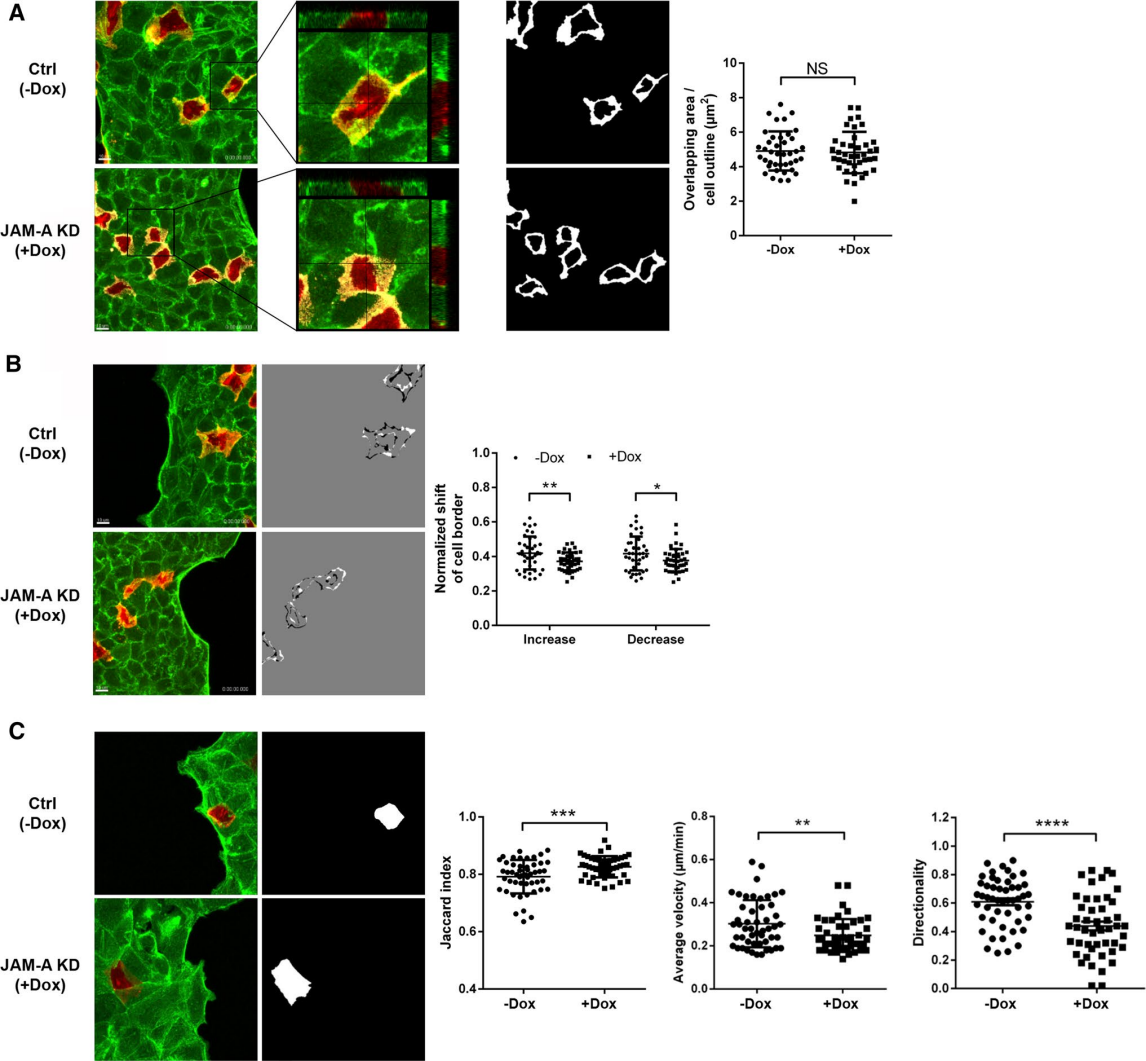
JAM-A regulates cryptic lamellipodia dynamics and cell motility in cellular collectives. **A** Formation of cryptic lamellipodia in collectively migrating cells. Homotypic suspensions of differently labeled (LA-mCherry and LA-EGFP, mixed at 1:5 ratio) MDCKII cells were seeded on microscope slides and observed by live cell microscopy over a period of 10 h. Left: representative immunofluorescence signals of cryptic lamellipodia. Higher power images of regions marked by black squares are shown adjacent to the immunofluorescence images. Middle: binary images of immunofluorescence images shown in the left panels. White areas highlight cryptic lamellipodia. Right: quantification of overlapping areas. Areas of overlapping regions were calculated on the basis of the merged LA-EGFP and LA-mCherry signals using an ImageJ Macro (see “Materials and Methods” for details). Data indicate the mean areas of overlap during the observation period. Each symbol represents the videomicroscopic analysis of one video. Number of videos analyzed: *n* = 40 for each condition. Data is derived from six independent experiments. Scale bars: 10 µm. **B** Dynamics of cryptic lamellipodia during collective cell migration. Left: representative immunofluorescence signals of overlapping areas. Middle: representative binary images illustrating increases (white areas) and decreases (black areas) of cellular overlaps over the observation period (10 h). Right: quantification of changes in overlapping regions. Changes were calculated using an ImageJ Macro on the basis of the merged LA-EGFP and LA-mCherry signals (see “Materials and Methods” for details). Data indicate the mean increase or decrease of overlapping regions during the observation period. Each symbol represents the videomicroscopic analysis of one video. Number of videos analyzed: *n* = 40 for each condition. Data are derived from six independent experiments. Scale bars: 10 µm. **C** Migration behavior of single cells embedded in a cell collective. Left: representative immunofluorescence signals of LA-mCherry-positive cells embedded in a LA-EGFP-positive collective. Middle: binary images of single LA-mCherry-positive cells. Right: Jaccard index, migration velocity and directionality of single cells. Note that high Jaccard index values reflect low motility (see “Materials and Methods” for details). Each symbol represents the video microscopic analysis of one individual cell. Number of cells analyzed: *n* = 50 for ctrl MDCKII cells, *n* = 47 for JAM-A KD MDCKII cells. Data is derived from six independent experiments. Statistical analysis of migration velocities was performed using Mann–Whitney *U* test, all other statistical analysis were performed using two-tailed Student’s *t* test. Data are presented as mean values ± SD. *NS* not significant; *P < 0.05, **P < 0.01, ***P < 0.001, ****P < 0.0001

To further understand the role of JAM-A during collective cell migration, we focused on single cells embedded in an environment of collectively migrating cells. For this, we performed collective cell migration experiments with mixed populations of LA-mCherry and LA-EGFP-labeled cells. We first analyzed the formation of cryptic lamellipodia by quantifying the extent of cellular overlap between individual cells and their neighbors on the basis of the merged mCherry and EGFP fluorescence signals. We observed that the total area of overlap between individual cells did not change after JAM-A depletion (Fig. 3A; Suppl. Movie 3, 4). However, JAM-A-depleted cells showed reduced protrusion dynamics as indicated by smaller areas of positive and negative protrusion formation, i.e., overlapping areas that grow or shrink over time (Fig. 3B, Suppl. Movie 5, 6). We next analyzed the motility, average velocity and directionality of individual cells within a collective of migrating cells over a period of 10 h. We observed that cell motility as analyzed by the relative change in a cell’s position was reduced after JAM-A depletion (Fig. 3C, Suppl. Movie 7, 8). Accordingly, the migration velocities as well as migration directionalities were reduced after JAM-A depletion (Fig. 3C). These findings indicated that JAM-A regulates the motility of individual cells during collective cell migration, most likely by impacting the dynamics of protrusion formation.

### JAM-A interacts with α3β1 integrin in MDCKII cells

**Fig. 4:**
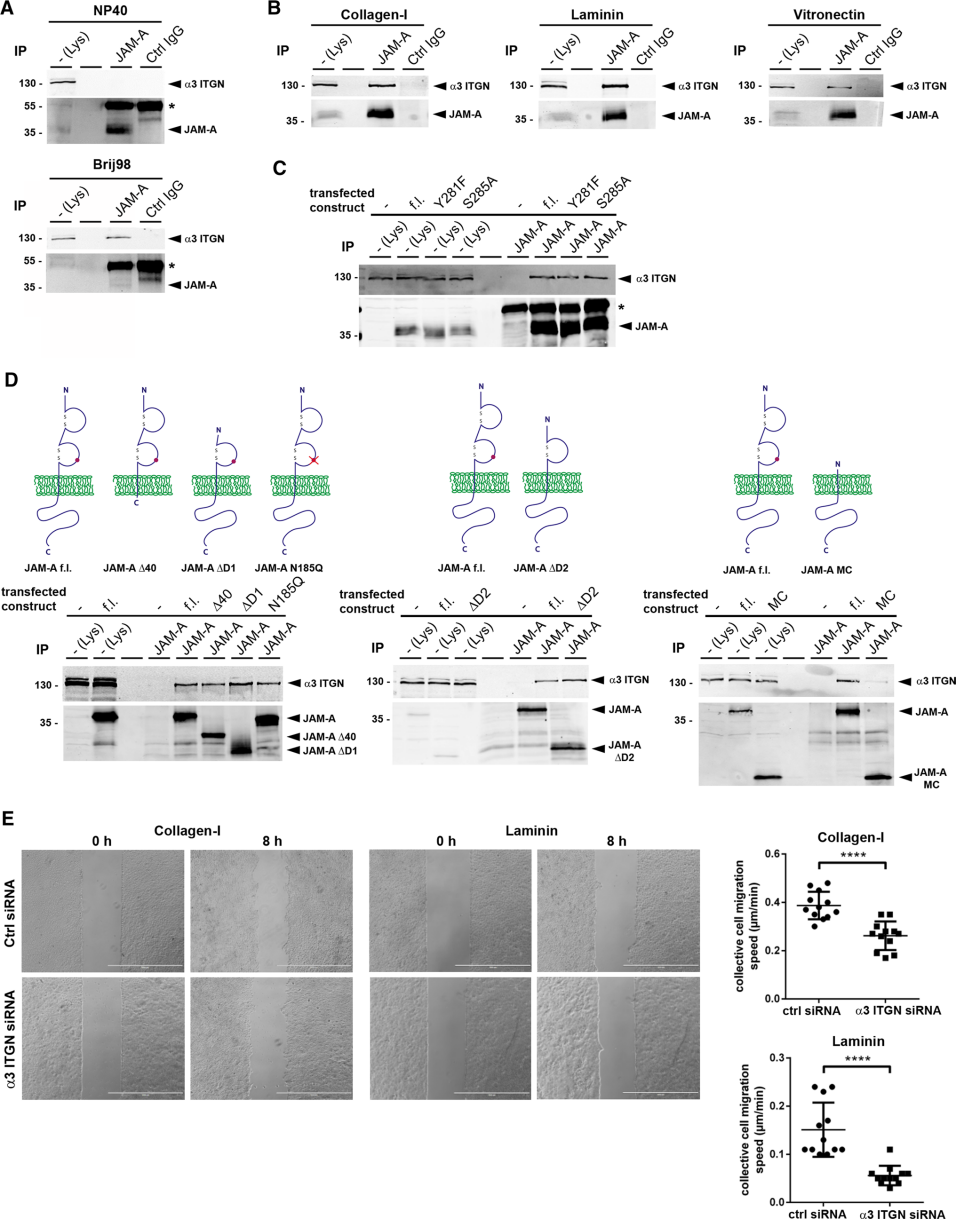
JAM-A interacts with α3β1 integrin in MDCKII cells. **A** CoIP of α3β1 integrin with JAM-A. The interaction of JAM-A with α3β1 integrin is detectable in Brij98-based lysates but not in NP40-based lysates. Note that the weak JAM-A signal in Brij98 lysed cells is due to lower solubility of JAM-A in Brij98. The asterisks indicate IgG heavy chains. *IP* immunoprecipitation, *Lys* lysate. **B** CoIP of α3β1 integrin with JAM-A from MDCKII cells cultured on collagen-I, laminin or vitronectin. JAM-A immunoprecipitates were immunoblotted for α3 integrin and JAM-A as indicated. To increase the solubility of JAM-A, the direct IPs for JAM-A were performed with NP40-based lysates. **C** CoIPs of JAM-A phospho-deficient mutants (JAM-A/Y281F, JAM-A/S285A) with α3β1 integrin. The asterisk indicates IgG heavy chains. **D** CoIPs of JAM-A mutants with α3β1 integrin. JAM-A constructs with deletions of the entire cytoplasmic domain (Δ40), the membrane-distal Ig-like domain (ΔD1), the membrane-proximal Ig-like domain (ΔD2), the entire extracellular domain (MC), or with point mutations affecting the N-linked glycosylation site of JAM-A (N185Q), were transfected into JAM-A-deficient MDCKII cells and analyzed for interaction with α3β1 integrin by CoIP. *f.l.* full length, *MC* membrane and cytoplasmic. **E** Collective cell migration velocity of control MDCKII cells (Ctrl siRNA pool) and α3 integrin KD MDCKII cells (α3 ITGN siRNA pool) on collagen-I and laminin. Left: representative images of monolayer expansion immediately after stamp removal (0 h) and after 8 h (8 h). Right: statistical evaluation of monolayer expansion on collagen-I and laminin. Each dot represents one independent cell population. Number of independent cell populations analyzed: *n* = 12 for each condition. Data are derived from three independent experiments. Statistical analysis shown in this figure was performed using two-tailed Student’s *t* test. Data are presented as mean values ± SD. ****P < 0.0001

The lack of efficient locomotion of cells during collective cell migration after depletion of JAM-A together with our observations that JAM-A-regulated single cell and collective cell migration depend on Col-I (Fig. 1A, Fig. 2A) suggested that JAM-A cooperates with a Col-I-binding integrin in MDCKII cells. We focused on α3 integrin, which forms a Col-I- and LN-binding heterodimer with β1 integrin [37, 38]. In co-immunoprecipitation (CoIP) experiments we found that JAM-A and α3β1 integrin exist in a complex in MDCKII cells (Fig. 4A). This interaction was detectable in Brij98-containing lysis buffer but not in NP40-containing lysis buffer (Fig. 4A). Since Brij98 maintains interactions mediated by tetraspanins (Tspans) [39], a family of integral membrane proteins with four membrane spanning domains [39–42], these findings suggested that the interaction between JAM-A and α3β1 integrin is indirect and mediated by a member of the Tspan superfamily. To test if the interaction depends on integrin activation by ligand binding, as described for the interaction between JAM-A and αvβ3 integrin in endothelial cells [26, 43], we performed CoIP experiments with MDCKII cells cultured on α3β1 integrin-interacting and -non interacting ECM proteins, i.e., LN and VN, respectively. The interaction was detectable on all ECM proteins (Fig. 4B) suggesting that JAM-A is constitutively associated with α3β1 integrin. To test if the interaction of JAM-A with α3β1 integrin depends on phosphorylation at Tyr281 or Ser285 of JAM-A, we analyzed the interaction of phosphorylation-deficient JAM-A mutants with α3β1 integrin. We observed no difference in the amount of α3β1 integrin associated with JAM-A/WT and JAM-A/Y281F and JAM-A/S285A (Fig. 4C), suggesting that phosphorylations of JAM-A do not mediate complex assembly.

To further characterize the interaction of JAM-A with α3β1 integrin we performed CoIP experiments with various mutants of JAM-A. The interaction of JAM-A with α3β1 integrin was detectable in the absence of the cytoplasmic domain (JAM-A/Δ40) indicating that the interaction involves the extracellular domain of JAM-A. The interaction was also detectable after deleting either of the two Ig-domains (JAM-A/ΔD1, JAM-A/ΔD2) as well as after mutating the N-linked glycosylation site (JAM-A/N185Q), but not after deleting the entire extracellular domain (JAM-A/MC) (Fig. 4D). These findings indicated that JAM-A interacts with α3β1 integrin through its extracellular domain.

To address the functional relevance of JAM-A–α3β1 integrin interaction, we analyzed collective cell migration of MDCKII cells after depletion of the α3 integrin subunit by RNAi. Similar to the depletion of JAM-A, depletion of the α3 integrin chain resulted in a reduced migration velocity of cell collectives grown on Col-I (Fig. 4E). Reduced migration velocity was also observed when cells were grown on LN (Fig. 4E), the second major ligand of α3β1 integrin [38]. These observations suggested that JAM-A and α3β1 integrin exist in a complex to cooperate in the regulation of collective cell migration in polarized epithelial cells.

### JAM-A interacts with the α3β1 integrin-interacting tetraspanin CD151

**Fig. 5:**
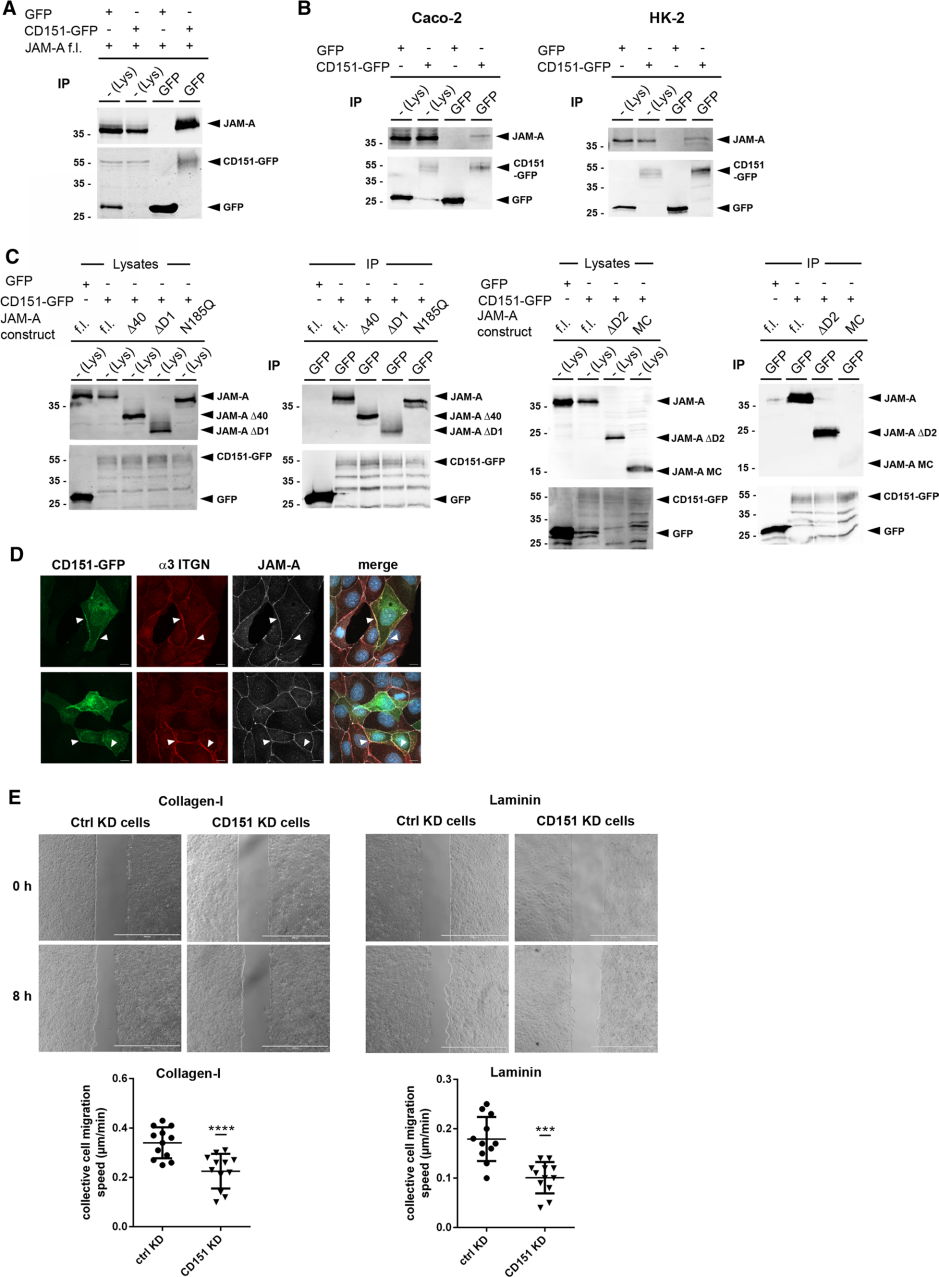
JAM-A interacts with tetraspanin CD151. **A** CoIP of CD151 with JAM-A in HEK293T cells. GFP-CD151 immunoprecipitates were immunoblotted for JAM-A (top panels) and GFP (bottom panels). **B** CoIP of CD151 with JAM-A in Caco2 cells (left) and HK-2 cells (right). GFP-CD151 immunoprecipitates were immunoblotted for JAM-A and GFP as indicated. **C** CoIPs of JAM-A deletion mutants with CD151. JAM-A mutant constructs (described in Fig. 4) were transfected into JAM-A-deficient MDCKII cells and analyzed for interaction with CD151 by CoIP. **D** IF analysis of MDCKII cells transfected with EGFP-tagged CD151 and immunostained for endogenous α3 integrin and JAM-A. Arrowheads indicate co-localization of CD151, α3 integrin and JAM-A at cell–cell contact sites. Scale bar: 10 µm. **E** Monolayer expansion assays of MDCKII cells after siRNA-mediated depletion of CD151. MDCKII cells were transiently transfected with siRNA pools directed against CD151 (CD151 KD cells). As control, MDCKII cells were transfected with a scrambled siRNA pool (Ctrl KD cells). Collective cell migration was analyzed as described in the legend to Fig. 2. Top: representative images of monolayer expansion immediately after stamp removal (0 h) and after 8 h (8 h). Collective cell migration was analyzed on collagen-I and laminin as indicated. Bottom: statistical evaluation of monolayer expansion on collagen-I and laminin. Each dot represents one independent cell population. Number of independent cell populations analyzed: *n* = 12 for each condition. Data are derived from three independent experiments. Statistical analysis was performed using two-tailed Student’s *t* test. Data are presented as mean values ± SD. ***P < 0.001, ****P < 0.0001

At least 12 different integrin heterodimers have been found in associations with Tspans [42, 44]. Tspans interact laterally with several binding partners including other Tspans, and Tspans frequently link integrins to members of the Ig superfamily [41, 42]. Since Tspan-mediated interactions are often sensitive to Triton X-100 or NP40-based lysis conditions [39, 41], our observation that the interaction of JAM-A with α3β1 integrin is sensitive to NP40-based lysis but detectable in Brij98-based lysis buffers suggested that the JAM-A–α3β1 integrin interaction is indirect and mediated by a member of the Tspan family. We focused on CD151/Tspan24, which directly interacts with α3β1 integrin through an association that involves the extracellular domains of the two proteins [45–47], and which regulates α3β1 function in many cell types [48–54]. We first ectopically expressed a EGFP-CD151 fusion protein in HEK293T cells and analyzed its interaction with JAM-A by CoIP. We found that JAM-A co-precipitated with EGFP-CD151 indicating that JAM-A and CD151 interact in cells (Fig. 5A). We also found that EGFP-CD151 interacted with endogenous JAM-A in two polarized epithelial cells, i.e., Caco2 cells and HK-2 cells (Fig. 5B). The interaction with CD151 was retained in the absence of the cytoplasmic domain but was lost when the entire extracellular domain was deleted (Fig. 5C) indicating that JAM-A interacts with CD151 through the same interface through which it interacts with α3β1 integrin. These observations suggested that JAM-A exists in a complex with CD151 and α3β1 integrin in which CD151 might link JAM-A to α3β1 integrin. When we analyzed the localization of this complex, we found that CD151 and α3β1 integrin localize to cell–cell contacts (Fig. 5D). A similar localization at cell–cell junctions has been observed before for CD151 and CD9 in MDCKII cells and in Caco2 cells [55]. Also, CD151 and CD9 have been described to co-localize with α3β1 integrin at intercellular junctions of keratinocytes [56]. These findings, thus, support the view that JAM-A interacts with CD151 and α3β1 integrin at intercellular junctions.

**Fig. 6:**
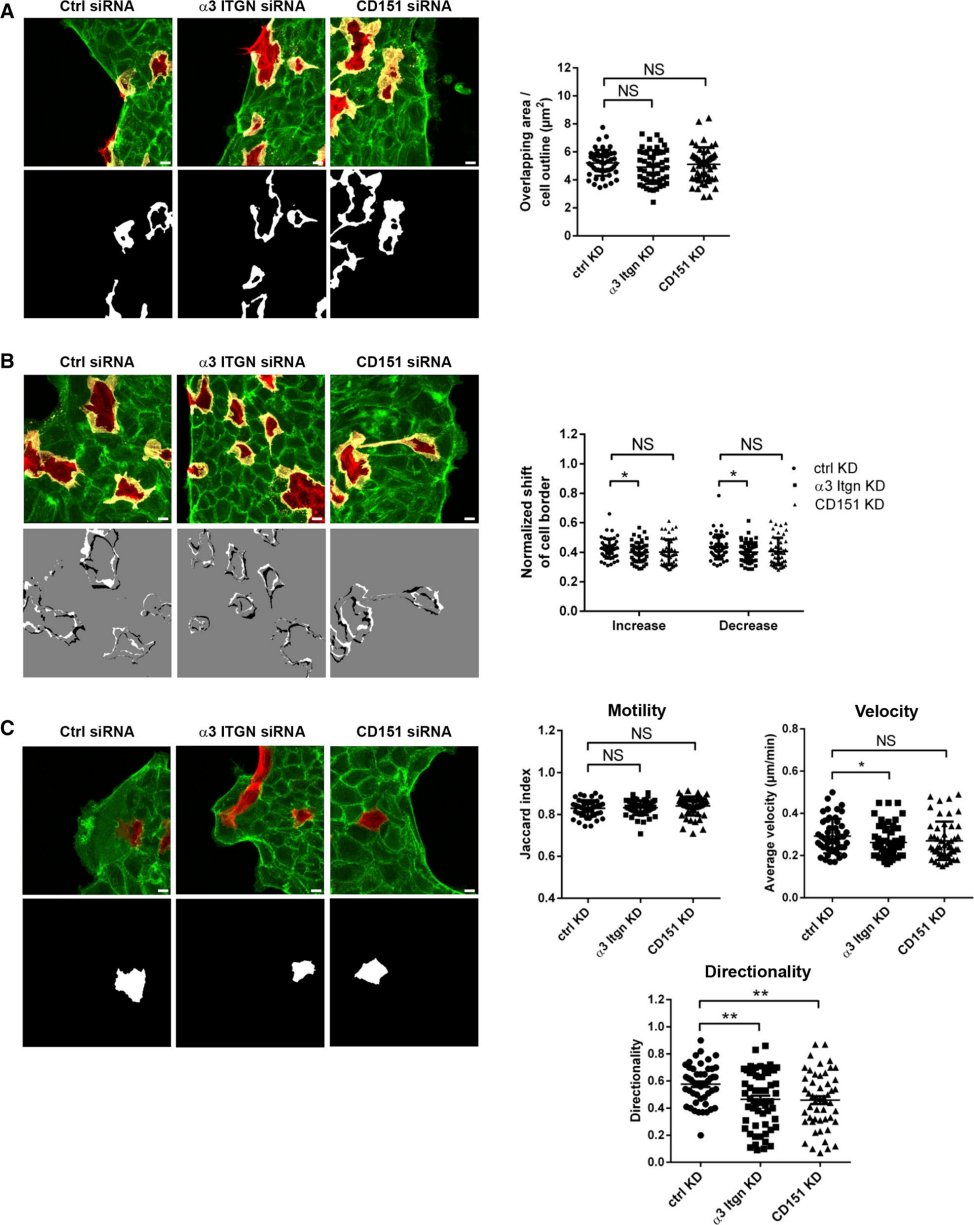
Regulation of cryptic lamellipodia dynamics and cell motility in cellular collectives by α3β1 integrin and Tspan CD151. **A** Formation of cryptic lamellipodia in α3 integrin- and CD151-depleted MDCKII cells. Note that the formation of cryptic lamellipodia is not altered after depletion of α3β1 integrin or of CD151. Scale bars: 10 µm. **B** Dynamics of cryptic lamellipodia during collective cell migration. Top panels: representative immunofluorescence signals of overlapping areas. Bottom panels: representative binary images illustrating increases (white areas) and decreases (black areas) of cellular overlaps over the observation period (10 h). Right panel: quantification of changes in overlapping regions. Changes were calculated using an ImageJ Macro on the basis of the merged LA-EGFP and LA-mCherry signals (see “Materials and Methods” for details). Data indicate the mean increase or decrease of overlapping regions during the observation period. Number of videos analyzed: n = 50, 10 independent experiments. Scale bars: 10 µm. **C** Migration behavior of single cells embedded in a cell collective. Left panels: representative immunofluorescence signals of LA-mCherry-positive cells embedded in a LA-EGFP-positive collective (top), and binary images of immunofluorescence images to highlight single LA-mCherry-positive cells. Right panels: quantification of motility, migration velocity and migration directionality of single cells within the collective. Note that high Jaccard index values reflect low motility (see “Materials and Methods” for details). Number of cells analyzed: *n* = 48 for ctrl MDCKII cells, *n* = 58 for α3 integrin KD MDCKII cells, *n* = 50 for CD151 KD MDCKII cells, 10 independent experiments. Scale bars: 10 µm. Statistical analysis of migration velocities was performed using Mann–Whitney *U* test; all other statistical analysis were performed using two-tailed Student’s *t* test. Data are presented as mean values ± SD. NS, not significant; *P < 0.05, **P < 0.01

To address the functional implication of CD151 during collective cell migration we performed monolayer expansion assays after RNAi-mediated depletion of CD151. We found that depletion of CD151 in MDCKII cells reduced the migration velocity of collectively migrating cells on both ECM ligands for α3β1 integrin, i.e., Col-I and LN (Fig. 5E). We also analyzed the behavior of single cells within sheets of collectively migrating cells after depletion of CD151 and after depletion of α3β1 integrin. While the dynamics of cryptic lamellipodia was slightly reduced after depletion of the α3 integrin chain but not after depletion of CD151, the directionality of migration was impaired by depletion of either CD151 or α3β1 integrin (Fig. 6). These observations indicate that both CD151 and α3β1 integrin contribute to collective cell migration by regulating the directionality of single cells within cellular collectives.

### JAM-A interacts with the α3β1 integrin-interacting tetraspanin CD9

**Fig. 7:**
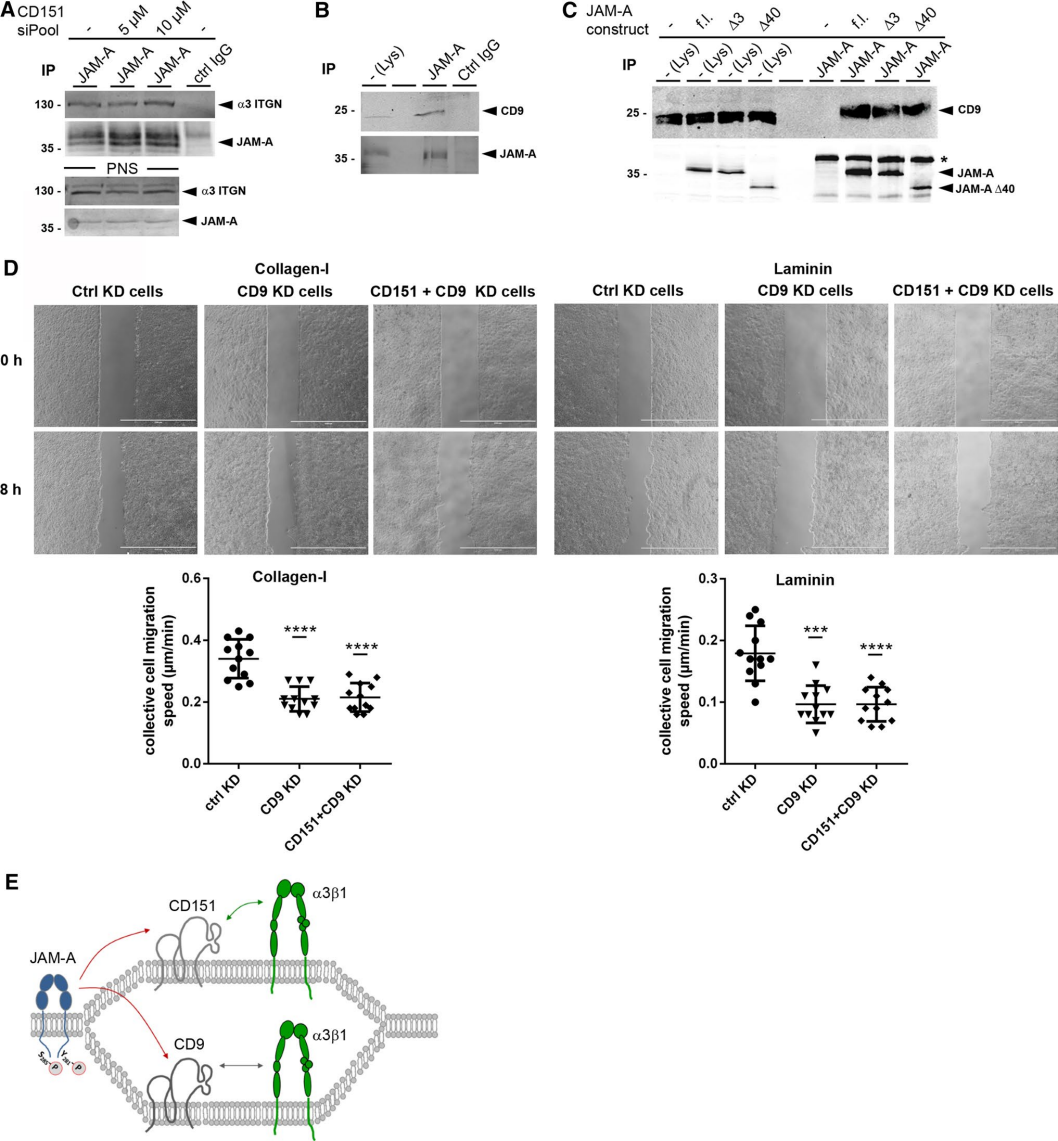
JAM-A interacts with tetraspanin CD9. **A** CoIP of α3β1 integrin with JAM-A in CD151-depleted MDCKII cells. Lanes with postnuclear supernatants (PNS) contain 5% of the input used for IPs. **B** CoIP of CD9 with JAM-A. JAM-A immunoprecipitates obtained from MDCKII cells were immunoblotted for CD9 (top panels) and JAM-A (bottom panels). **C** CoIPs of JAM-A deletion mutants with endogenous CD9. JAM-A deletion constructs lacking the PDZ domain-binding motif (Δ3) or the entire cytoplasmic domain (Δ40) were transfected into JAM-A-deficient MDCKII cells and analyzed for interaction with CD9 by CoIP. Note that the JAM-A–CD9 interaction is retained after deletion of the entire cytoplasmic domain. The asterisk indicates IgG heavy chains. (**D**) Monolayer expansion assays of MDCKII cells after siRNA-mediated depletion of CD9 and after combined depletion of CD151 and CD9. MDCKII cells were transiently transfected with siRNA pools directed against CD9, or both CD151 and CD9. As control, MDCKII cells were transfected with a scrambled siRNA pool (Ctrl KD cells). Collective cell migration was analyzed as described in the legend to Fig. 2. Top: representative images of monolayer expansion immediately after stamp removal (0 h) and after 8 h (8 h). Collective cell migration was analyzed on collagen-I and laminin as indicated. Bottom: statistical evaluation of monolayer expansion on collagen-I and laminin. Each dot represents one independent cell population. Number of independent cell populations analyzed: *n* = 12 for each condition. Data are derived from three independent experiments. Statistical analysis was performed using two-tailed Student’s *t* test. Data are presented as mean values ± SD. ***P < 0.001, ****P < 0.0001. The experiments were performed in parallel to the monolayer expansion assays shown in Fig. 5E. The data obtained for control cells are therefore identical to those shown in Fig. 5E. **E** Cartoon of JAM-A association with α3β1 integrin through tetraspanins CD151 and CD9 (summary of biochemical data). Note that the interaction of JAM-A with both tetraspanins is mediated by the extracellular domain of JAM-A (red arrows), and that the interaction of CD151 with α3β1 integrin is also mediated by the extracellular domains of the two proteins (green arrow) [45]. Phosphorylations involved in the regulation of MDCK cell motility and collective cell migration are indicated. For simplicity, the two phosphorylations are depicted in the same JAM-A dimer. Western blot data shown in this figure are representatives of at least three independent experiments

Since TEMs frequently contain more than one Tspan protein [39], we tested if CD151 is solely responsible for the JAM-A—α3β1 integrin association. We first performed CoIP experiments in CD151-depleted MDCKII cells and found that the JAM-A—α3β1 integrin interaction was retained in the absence of CD151 (Fig. 7A), suggesting the presence of additional Tspans in the JAM-A–CD151–α3β1 integrin-containing TEM, or alternatively, the existence of a CD151-independent JAM-A–α3β1 integrin-containing TEM. To identify a potential Tspan family member that could link JAM-A to α3β1 integrin, we focused on CD9/Tspan29, which has been described as a second α3β1 integrin-interacting Tspan [42, 57], and also as a JAM-A-interacting Tspan in platelets and endothelial cells [26, 58]. By CoIP we found that CD9 interacts with JAM-A in MDCKII cells (Fig. 7B). This interaction was retained in the absence of the cytoplasmic domain of JAM-A (Fig. 7C) indicating that in contrast to endothelial cells, in which the interaction with CD9 is mediated through its PDZ domain-binding motif [26], JAM-A interacts with CD9 in MDCK epithelial cells through its extracellular domain. These findings suggested that the physical interaction of JAM-A with α3β1 integrin can also be mediated by Tspan CD9. To address a functional relevance of the JAM-A–CD9 interaction, we performed monolayer expansion assays after RNAi-mediated depletion of CD9 in MDCKII cells. Depletion of CD9 resulted in a similar reduction of migration velocity of collectively migrating cells as observed after depletion of JAM-A (Fig. 7D). Overall, these findings suggest that JAM-A is linked to α3β1 integrin in a TEM (or in separate TEMs) by Tspans CD151 and CD9 to form a functional unit with α3β1 integrin which regulates collective cell migration in polarized epithelial cells. A cartoon summarizing the interaction of JAM-A with α3β1 integrin through Tspans CD151 and CD9 is depicted in Fig. 7E.

## Discussion

JAM-A is a cell–cell adhesion receptor that is localized at cell–cell junctions of polarized epithelial cells with enrichment at the TJs [18, 59, 60]. Its role in the regulation of the epithelial barrier function, which involves phosphorylation at two sites in the cytoplasmic tail, has been intensively studied [18, 27, 29, 60]. By addressing JAM-A’s role in cell migration of polarized epithelial cells, we find that JAM-A limits cell motility when cells migrate as individuals but promotes cell migration when cells migrate as a cellular sheet. The regulation of collective cell migration is most likely mediated through its interaction with α3β1 integrin, which is mediated by Tspans CD151 and CD9. We propose the existence of a TEM in which Tspans CD151 and CD9 connect JAM-A to α3β1 integrin to assemble a signaling complex that allows a functional crosstalk of JAM-A and α3β1 integrin during collective cell migration.

### Cell motility and collective cell migration

One observation in our study is that JAM-A depletion results in increased motility of single cells but reduced motility when cells are embedded in a collective of migrating cells. These observations, thus, indicated a cell-autonomous function of JAM-A in single epithelial cells. A similar, i.e., cell-autonomous and motility-limiting function of JAM-A has been observed in endothelial cells and dendritic cells (DCs) [61, 62]. In JAM-A-deficient endothelial cells the increased motility correlates with longer protrusions [62], suggesting that JAM-A limits protrusion formation in these cells. This activity has been proposed to be mediated through glycogen synthase kinase (GSK)-3β and its upstream regulator atypical protein kinase C-zeta (aPKCζ). In MDCK cells, GSK-3β regulates membrane protrusions and migration through Rac1 [63] opening the possibility that JAM-A regulates single cell motility in polarized epithelial cells through a similar mechanism as in endothelial cells. Interestingly, JAM-A has also been found to regulate cell motility of neutrophils. In these cells, JAM-A co-clusters with α5β1 integrin and regulates β1 integrin internalization and recycling, and the absence of JAM-A results in increased β1 integrin surface localization concomitant with reduced cell motility [64]. Our findings of an increased motility of singly migrating MDCKII cells adds to the growing evidence of a cell-autonomous function of JAM-A.

Surprisingly, despite an increased motility of singly migrating cells after JAM-A depletion, individual cells embedded in a collective of cells show an impaired motility, reduced migration velocity and reduced directionality after JAM-A depletion, which results in a reduced migration velocity of the cell collective. We found that the size of the cryptic protrusions does not change upon JAM-A depletion. However, the dynamics of cryptic lamellipodia formation is reduced, which is expected to result in more static cell–matrix interactions and, thus, reduced motility of single cells. We speculate that JAM-A depletion alters traction force generation. JAM-A depletion in MDCK cells has recently been found to enhance focal adhesion formation and to increase traction forces [65]. An enhanced formation of focal adhesions resulting in stronger cell–matrix adhesion and concurrent increased traction forces would explain why JAM-A-depleted MDCK cells show reduced migration velocities during collective cell migration [66]. Since we observed a reduced migration velocity upon JAM-A depletion when cells migrate as collective but not when cells migrate as single cells, we speculate that the regulation of traction force formation by JAM-A depends on signaling events triggered by trans-homophilic interactions of JAM-A.

An intriguing possibility how JAM-A-generated signaling events influences cell–matrix adhesions and traction force generation during collective cell migration is that JAM-A acts as a sensor of mechanical forces during collective cell migration. Studies with collectives of migrating cells both in vitro and in vivo indicate that collective cell migration requires mechanical coordination between neighboring cells, and that mechanical forces are transmitted through cell–cell contacts from leader cells at the edges of migrating colonies to the follower cells in their center over a long range [11–13, 15, 67], reviewed in [68, 69]. Tension on JAM-A applied through trans-homophilic JAM-A interaction activates RhoA in a Ser285 phosphorylation-dependent manner in CHO cells [70]. Importantly, in MDCK cells JAM-A regulates tensile stress on the TJ scaffolding protein ZO-1 through its interaction with PDZ domain 3 of ZO-1 [65, 71]. These observations, thus, suggest the possibility that JAM-A localized at TJs contributes to the regulation of collective cell migration by transmitting tensile forces at the TJs to its directly associated scaffold protein ZO-1 which changes conformation in response to tensile forces [65, 72]. This possibility is supported by our observation that Ser285 phosphorylation of JAM-A, which is observed exclusively at the TJs [18], is required for the regulation of collective cell migration of JAM-A (Fig. 2). JAM-A might, thus, contribute to the regulation of collective cell migration by different mechanisms, one involving the transmission of mechanical strain through TJs and concurrent regulation of actomyosin contractility, traction forces and focal adhesion formation via RhoA [65, 72, 73], and one involving the regulation of integrin-dependent functions through its association with α3β1 integrin (see below).

### The JAM-A–CD151–α3β1 TEM

As the second major observation of our study, we find that JAM-A interacts with α3β1 integrin in MDCK cells. This interaction involves the extracellular domain of JAM-A, identifying for the first time a cis-heterophilic interaction partner which is linked to the extracellular domain of JAM-A. This interaction is most likely indirect and mediated by a member of the Tspan family, since it is only detectable under lysis conditions that retain Tspan-mediated interactions, i.e., Brij98 [39, 42]. We identified Tspans CD151 and CD9 as possible mediators of this interaction. Both tetraspanins are binding partners of α3β1 integrin [42]. We consider CD151 as more likely to link JAM-A and α3β1 integrin during collective cell migration since CD151 has been found to regulate a number of α3β1 integrin-dependent functions including cell–matrix adhesion, cell motility and migration on LN [47, 51, 54, 74], intercellular junction formation [49, 53, 75] or cell survival [76]. Our observations that depleting either α3β1 integrin or CD151 or CD9 results in a similar retardation of collective cell migration in MDCK cells like depleting JAM-A supports the view that JAM-A regulates collective cell migration of MDCK cells through its physical association with α3β1 integrin mediated by CD151 and CD9.

How JAM-A regulates collective cell migration through these associations remains unclear. Both α3β1 integrin and CD151 have been identified at cell–cell junctions of various cell types including tumor cells [53, 75], keratinocytes [56, 76], and kidney epithelial cells derived from collecting ducts [49]. Therefore, it is likely that the CD151 (and possibly CD9)-based TEM containing JAM-A and α3β1 integrin is localized at cell–cell junctions. We speculate that JAM-A regulates α3β1 integrin-associated functions. Studies in platelets have shown that Tyr-phosphorylated JAM-A recruits the Src kinase Csk to inhibit αIIbβ3-associated Src during outside-in signaling [77]. We therefore envisage a scenario for MDCK cells in which the lateral association of JAM-A with α3β1 integrin through a tetraspanin as linker protein might serve to generate spatial proximity between JAM-A and possibly a JAM-A-associated regulatory protein and an α3β1 integrin-associated signaling molecule. Interestingly, we found that a JAM-A construct with a mutation in the known Tyr phosphorylation site (JAM-A/Y281F) [29] fails to restore single cell and collective cell migration in JAM-A null MDCK cells to levels observed after expression of JAM-A/WT (Figs. 1, 2), strongly suggesting that Tyr281 phosphorylation is required to mediate JAM-A’s function during single and collective cell migration. This observation is intriguing since phosphorylation/dephosphorylation allows for dynamic regulation of signaling events as they occur during cell migration. Recent observations in integrin-based cell–matrix adhesion complexes indicate that signal propagation within these complexes does not involve gross changes in the composition of the adhesion complex, but is regulated by the relay of phosphotyrosine-dependent signals [78]. The incorporation of JAM-A and α3β1 integrin in a single protein cluster by the activity of CD151 and possibly CD9 could, thus, serve to rapidly transmit phospho-JAM-A-dependent regulatory events on α3β1 integrin-dependent signaling pathways.

## Materials and methods

### Cell culture and transfections

MDCKII cells with inducible expression of JAM-A shRNAs [27] or CRISP/Cas9-mediated inactivation of the JAM-A gene [28], and HEK293T cells (ATCC #CRL-3216) were cultivated in DMEM high glucose medium (Sigma-Aldrich (SA) #D5671) containing 10% FCS, 2 mM glutamine, 100 U/ml penicillin and 100 U/ml streptomycin. For stable MDCKII cell lines with inducible expression of JAM-A shRNAs, the growth media were additionally supplemented with 500 μg/ml G418 and 5 μg/ml blasticidin [79], and shRNA expression was induced by the addition of 2 µg/ml doxycycline. Stable LifeAct-EGFP (LA-EGFP) or LifeAct-mCherry (LA-mCherry) expressing MDCKII cells were generated by lentiviral transduction and selection in growth media containing 0.1 mg/ml zeocin or 1 µg/ml puromycin, respectively. MDCKII JAM-A KO cell lines expressing murine JAM-A (mJAM-A)/WT, mJAM-A/Y281F or mJAM-A/S285A under a tetracycline-regulated promotor were generated by lentiviral transduction and maintained in DMEM media containing 1 µg/ml puromycin and 2 µg/ml doxycycline (selectively added for induction of mJAM-A expression). These cell lines were additionally transfected with LA-EGFP or LA-mCherry, as described above. Cell–cell contact integrity in JAM-A KO MDCKII cells was verified by immunofluorescence microscopy for ZO-1 (Suppl. Fig. S1B). The expression levels as well as the subcellular localization of the ectopically expressed JAM-A constructs are shown in Suppl. Fig. S1C.

Transient transfections of siRNAs were performed using Lipofectamine RNAiMAX (Thermo Fisher Scientific). Transfections of expression vectors were performed using Lipofectamine 2000 (Thermo Fisher Scientific) or Xfect transfection reagent (TaKaRa Bio Europe SAS, Saint-Germain-en-Laye, France) according to the manufacturer's instructions. Lentiviral particles for the generation of stably transfected cell lines expressing either shRNAs or cDNAs were generated by cotransfection of the lentiviral vector and the packaging vectors psPAX2 and pMD2.G (kindly provided by Dr. Didier Trono, Addgene plasmids 12,260 and 12,259) in a ratio of 3:2:1 into HEK293T cells. Lentiviral transduction of cells was performed as described [79].

### RNA interference, plasmid vectors and constructs

The following siRNAs and shRNAs directed against canine (c) genes were used: cJAM-A shRNA 5′-CCAGTAAGAAGGTGATTTA-3′ (in pLVTHM (Addgene #12247) and in pEmU6proT (provided by Dr. Karl Matter); cCD151 siRNA pool (NCBI Gene ID475992, siTOOLs Biotech, München), cCD9 siRNA (NCBI Gene ID611695, siTOOLs Biotech, München), cITGA3 (NCBI Gene ID491074, siTOOLs Biotech, München); negative control siRNA pool (neg. ctrl. siPOOL Neg D1, siTOOLs Biotech, München); negative control siRNA (Qiagen 1027280), OnTarget plus non-targeting pool (Dharmacon D-001810-10-05). Knockdown efficiencies of all shRNAs and siRNAs used in this study are depicted in Suppl. Figs S1A, S1D, S1E. The following constructs were used: LifeAct-eGFP in pFUGW, LifeAct-mCherry in pLV-PGK-Puro (provided by Dr. H. Farin); hJAM-A/WT, hJAM-A/Δ3, hJAM-A/Δ40, ΔD1-hJAM-A, hJAM-A/MC, hJAM-A/N185Q in pFLAG-CMV-1 (SA, Munich, Germany); Flag-mJAM-A/WT, Flag-mJAM-A/Y281F, Flag-mJAM-A/S285A in pInducer21-Puro (Addgene #46948) [80] (provided by Dr. T. Weide); hCD151 in pEGFP-C1 (TaKaRa Bio).

### Antibodies and reagents

The following antibodies were used in this study: mouse mAb anti-α-catenin (BD TL 610194, IF 1:600), mouse mAb anti-β-catenin (BD TL 610154, IF 1:600), mouse mAb anti-p120^ctn^ (BD TL 610134, IF 1:600), mouse mAb anti-E-cadherin (BD TL 610181, IF 1:600), mouse mAb anti-Paxillin (BD TL 610,051, WB 1:500, IF 1:600), mouse mAb anti-CD9 clone MM2-57 (Millipore CBL162, WB 1:1000), rabbit pAb anti-Flag (SA, F7425, IF 1:1000, WB 1:1000), rabbit pAb anti-GAPDH clone FL-335 (SCBT SC-25778, WB 1:3000), mouse mAb anti-Integrin α3/CD49c (BD TL 611044, WB 1:500, IF 1:500), mouse mAb anti-Integrin β1 / CD29 (BD TL 610467, WB 1:500), mouse mAb anti-α-tubulin (SA, clone B-5-1-2, T5168, WB 1:10,000), rat mAb anti-ZO-1 (kindly provided by Dr. B. Stevenson, University of Alberta, Edmonton, Canada), clone R40.76, IF 1:800). Isotype-specific control antibody: rabbit IgG (Thermo Scientific 02-6102). A polyclonal antibody against canine JAM-A (Affi831) was generated by immunizing rabbits with a fusion protein consisting of the extracellular part of canine JAM-A fused to the Fc-part of human IgG, as described previously [27]. Reagents: recombinant human vitronectin (VN, Peprotech 140-09), rat tail type I collagen (Col-I, Advanced BioMatrix #5153), laminin (LN, isolated from human fibroblasts, SA #L4544), fibronectin (FN, isolated from human plasma, SA #F2006).

### Quantitative polymerase chain reaction (qPCR)

To analyze the mRNA level of CD9 and CD151, the total RNA was extracted from the cell lysate using the RNeasy Mini Kit following the instructions from Qiagen (Hilden, Germany) and transcribed in cDNA using the High-Capacity cDNA Reverse Transcription Kit from Applied Biosystems (Waltham, Massachusetts, US). The following primer pairs were used (forward; reverse): CD9_1: GCTCTTGCTGGGATTGCAG; GAAACCCACCAGCATCATG, CD9_2: CTGGGGTTGTTCTTTGGC; CGTAGTGGATGGCCTTCAG, CD9_3: GTGGAACAGTTTATCTCCGAC; GATAGCACAGCACAGGATC, CD151: GAGAAGACGACATGTGGCAC; GTAGGCTGTGGCCAGGTAG, CD151_2: GTTGTCGTCATGGTGACTG; GTTCAGCTGCTGGTAGTAG, CD151_3: CAACCTGAAGGACACCATG; CACCCTCCACCTTGTAGATG, GAPDH: TCACCACCATGGAGAAGGC; GGCTAGAGGAGCCAAGCAG, HPRT: GCTTGCTGGTGAAAAGGAC; TTATAGTCAAGGGCATATCC.

### Immunoprecipitation and western blot analysis

For immunoprecipitations, cells were lysed in lysis buffer (150 mM NaCl, 1 mM CaCl_2_, 1 mM MgCl_2_, 0,02% (w/v) NaN_3_, 10 mM TrisHCl pH 7.4, 1% (w/v) Brij98 (Merck, Darmstadt, Germany) supplemented with protease inhibitors (Complete Protease Inhibitor Cocktail; Roche, Indianapolis, IN) and phosphatase inhibitors (PhosSTOP™, Roche, Indianapolis) for 30 min on ice and then centrifuged for 20 min at 4 °C. For one experiment (Fig. 4A), 1% (v/v) Nonidet P-40 (NP-40, AppliChem, Darmstadt, Germany) was used as an alternative for Brij98. The resulting supernatants were incubated with 2.5 µg of antibodies coupled to protein A-Sepharose beads (GE Healthcare, Solingen, Germany) for 4 h at 4 °C. Afterwards, the beads were washed five times with lysis buffer and bound proteins were eluted using SDS sample buffer. For Western blot analysis of the tetraspanin CD9 by immunoblotting, SDS sample buffer without DTT was added for elution, and 50 mM DTT was selectively added to the samples used for the detection of JAM-A. Proteins were separated by SDS-PAGE and analyzed by Western blot with near-infrared fluorescence detection (Odyssey Infrared Imaging System Application Software Version 3.0 and IRDye 800CW- or IRDye 680CW-conjugated antibodies; LI-COR Biosciences, Bad Homburg, Germany). All immunoprecipitations shown in the figures are representatives for at least three experiments. For quantification of signal intensities, the same software as for data acquisition (Odyssey application software Version 3.0) was used.

### Immunoprecipitation of GFP fusion proteins

GFP fusion proteins were precipitated by using the GFP Selector (NanoTag Biotechnologies, Göttingen, Germany). After cell lysis, the postnuclear supernatant was added to 20 µl of GFP Selector slurry. After incubation for 1 h at 4 °C, the GFP Selector beads were washed five times with lysis buffer. Bound proteins were eluted by boiling in SDS sample buffer analyzed by Western blotting as described above.

### Immunofluorescence microscopy

For immunofluorescence microscopy, cells were grown on collagen-I-coated glass slides for 24 h and subsequently fixed with 4% paraformaldehyde (PFA, Sigma-Aldrich) for 10 min at room temperature (RT) or methanol (BioChemica, Billingham, England) for 5 min on ice. For PFA-fixed cells, permeabilization was performed by applying 0.5% Triton X-100 in PBS for 10 min at RT and the cells were washed three times with 100 mM glycine in PBS. For both fixation methods, unspecific binding sites were blocked for 1 h using blocking buffer (10% FCS, 0.2% Triton X-100, 0.05% Tween-20, 0.02% BSA in PBS) and the primary antibody was applied overnight at 4 °C. After washing three times with PBS, the cells were incubated with fluorochrome (AlexaFluor488, AlexaFluor594 or AlexaFluor647)-conjugated, highly cross-adsorbed secondary antibodies (Invitrogen) and 2,4,diamidino-2-phenylindole (DAPI, Sigma-Aldrich) for 2 h at RT. F-actin was visualized using AlexaFluor647-conjugated phalloidin. The samples were washed thoroughly with PBS and mounted in fluorescence mounting medium (Mowiol 4-88, Sigma-Aldrich). Immunofluorescence microscopy was performed using the confocal LSM800 Airyscan microscope (Carl Zeiss, Jena, Germany) equipped with a Plan-Apochromat × 63/1.4 oil differential interference contrast objective (Carl Zeiss). Image processing and quantification was performed using ZEN 2012, ImageJ (National Institutes of Health, Bethesda, MD) and Imaris (Bitplane, Version 9.1.2) software.

### Single cell migration

For single cell migration assays, MDCK II cells transfected with either LA-EGFP or LA-mCherry were seeded on  Col-I-, VN-, FN-, or LN-coated microscope slides (Ibidi µ-Slide 8 well glass bottom, Ibidi #80827) at a cell density of 2000 cells / cm^2^. After overnight incubation, the cells were analyzed using the confocal LSM780 microscope (Carl Zeiss, Jena, Germany) with a Plan-Neofluar × 20/0.5 objective for 10 h with images taken every 10 min. The velocity of single cells was determined by tracking the cells using the TrackMate Plugin from ImageJ, which tracks the cell from frame to frame and thereby determines its average velocity.

### Collective cell migration

For the analysis of collective cell migration in epithelial monolayers, a monolayer expansion assay was used in which collective cell migration is triggered by a free surface [13, 32]. MDCK cells were seeded in different compartments of Col-I-, VN-, FN-, or LN-coated microscope slides (Ibidi µ-Slide 2 well glass bottom, Ibidi #80287) separated by a removable silicone stamp (Ibidi Culture-Inserts 2 Well for self-insertion, Ibidi #80209). Cells were grown for 72 h to confluency before removal of the stamp to trigger sheet migration. Pictures were taken directly after removal of the stamp and 8 h later using an EVOS digital inverted microscope. The collective cell migration speed was calculated as the mean distance of the front of the cell sheet from the initial position of the cell sheet’s front at the end of the observation time using ImageJ. Briefly, the cell-free area measured at the end of the observation period (*t*_1_ = 8 h) was subtracted from the cell-free area at the beginning (*t*_0_) resulting in the total area covered by migrated cells. This area was divided by the height of the gap resulting in the total distance that the cells had migrated. To take into account that the cells close the gap from both sides, the total distance was divided by two resulting in the distance migrated by a single sheet. The migration speed of the cellular collective was calculated by dividing the distance of a single sheet by the observation time and is given in µm/min. Experiments were performed at least three times with at least three separate migration chambers (biological replicates) per experiment.

### Analysis of single cell motility within cell collectives

Cell motility of single cells embedded in migrating cell collectives was analyzed by seeding co-cultures of LA-mCherry- and LA-EGFP-transfected MDCK cells (mixed at ratios of 1:5) on Col-I-coated microscope slides used for collective cell migration (see above). After cells had reached full confluency (72 h after seeding) stamps were removed to induce collective cell migration. 6 h after removal of the stamps, cell behavior was recorded for 10 h with images taken every 3 min using the confocal LSM780 microscope (Carl Zeiss, Jena, Germany) with a Plan-Apochromat × 63/1.4 oil objective. For analysis, the LA-mCherry and LA-EGFP channels, a median filter was applied and cells from one channel were segmented as binary (black and white) images (BW) by manual thresholding.

For single cells movies, cell segmentation was performed according to [81]. Briefly, the center of the segmented cell was determined for each frame to calculate the average velocity and directionality using the TrackMate Plugin for ImageJ. To access the dynamic changes of the cell outline, the segmentations of a cell at two different time points was compared using the Jaccard index *J* according to the following formula:

$$J\left(t\right)=\frac{\left|A\cap B\right|}{\left|A\cup B\right|},$$

where *A* and *B* are the segmented cells at time point $(t-\Delta t)$ and $(t+\Delta t)$, respectively:

$$A=BW\left(t-\Delta t\right),$$

$$B=BW\left(t+\Delta t\right).$$

The Jaccard index is equal to 1 if the cell outline is not changed between the two time points and tends to zero for higher differences, indicating higher protrusion/retraction activity at the cell border. Note, that this index can change, even if the center of the segmented cell does not move at all.

For movies with one or more cells, the protrusion and retraction activity along the segmented region was quantified. To estimate the protrusion activity, the area that was exclusively occupied by the segmented cells at the time point $(t+\Delta t)$ but not at time point $(t-\Delta t)$ was determined and normalized to the perimeter of the segmented area at time point *t*:

$$Area increase\left(t\right)=\frac{|B\backslash A|}{perimeter(BW\left(t\right))}.$$

In analogy, the retraction activity was calculated:

$$Area decrease\left(t\right)=\frac{|A\backslash B|}{perimeter(BW\left(t\right))}.$$

For these calculations, $\Delta t$ was set to 3 min.

All parameters are represented as mean values over the entire duration of the experiment. The scripts to calculate the Area Increase, the Area Decrease and the Jaccard indices are freely available by the authors upon request.

### Statistics

Results are expressed as arithmetic means ± SD as indicated. To test the normality of data, D’Agostino–Pearson normality test was used. Data with normal distributions were statistically compared by using unpaired, two-tailed Student’s *t* test, whereas Mann–Whitney *U* test was applied for data without normal distribution. Statistical analyses were performed using GraphPad Prism version 6 (GraphPad Software, San Diego, CA). *P* values are indicated as follows: *P < 0.05, **P < 0.01, ***P < 0.001 and ****P < 0.0001.

## Supplementary Information

Below is the link to the electronic supplementary material.

Supplementary file1 (PDF 764 KB)

Supplementary file2 (AVI 1116 KB)

Supplementary file3 (AVI 1087 KB)

Supplementary file4 (AVI 12513 KB)

Supplementary file5 (AVI 12897 KB)

Supplementary file6 (AVI 14447 KB)

Supplementary file7 (AVI 10935 KB)

Supplementary file8 (AVI 12901 KB)

Supplementary file9 (AVI 14047 KB)

## Abbreviations

Col-I: Collagen type I
ECM: Extracellular matrix
FN: Fibronectin
JAM: Junctional adhesion molecule
LA: LifeAct
LN: Laminin
TEM: Tetraspanin-enriched microdomain
Tspan: Tetraspanin
VN: Vitronectin

## Declarations

### Availability of data and material

All data generated or analyzed during this study are included in this published article and its supplementary information files, or are available from the corresponding author upon reasonable request.

### Code availability

Software scripts are freely available from the authors upon request.

## References

[1] Friedl P, Gilmour D (2009). Collective cell migration in morphogenesis, regeneration and cancer. Nat Rev Mol Cell Biol.

[2] Scarpa E, Mayor R (2016). Collective cell migration in development. J Cell Biol.

[3] Shellard A, Mayor R (1807). Rules of collective migration: from the wildebeest to the neural crest. Philos Trans R Soc Lond B Biol Sci.

[4] Farooqui R, Fenteany G (2005). Multiple rows of cells behind an epithelial wound edge extend cryptic lamellipodia to collectively drive cell-sheet movement. J Cell Sci.

[5] Menko AS, Bleaken BM, Walker JL (2014). Regional-specific alterations in cell-cell junctions, cytoskeletal networks and myosin-mediated mechanical cues coordinate collectivity of movement of epithelial cells in response to injury. Exp Cell Res.

[6] Ozawa M (2020). Adherens junction regulates cryptic lamellipodia formation for epithelial cell migration. J Cell Biol.

[7] Abu Taha A (2014). ARP2/3-mediated junction-associated lamellipodia control VE-cadherin-based cell junction dynamics and maintain monolayer integrity. Mol Biol Cell.

[8] Hayer A (2016). Engulfed cadherin fingers are polarized junctional structures between collectively migrating endothelial cells. Nat Cell Biol.

[9] Cao J (2017). Polarized actin and VE-cadherin dynamics regulate junctional remodelling and cell migration during sprouting angiogenesis. Nat Commun.

[10] Theveneau E (2010). Collective chemotaxis requires contact-dependent cell polarity. Dev Cell.

[11] Tambe DT (2011). Collective cell guidance by cooperative intercellular forces. Nat Mater.

[12] Cai D (2014). Mechanical feedback through E-cadherin promotes direction sensing during collective cell migration. Cell.

[13] Das T (2015). A molecular mechanotransduction pathway regulates collective migration of epithelial cells. Nat Cell Biol.

[14] Plutoni C (2016). P-cadherin promotes collective cell migration via a Cdc42-mediated increase in mechanical forces. J Cell Biol.

[15] Sunyer R (2016). Collective cell durotaxis emerges from long-range intercellular force transmission. Science.

[16] Bazellieres E (2015). Control of cell-cell forces and collective cell dynamics by the intercellular adhesome. Nat Cell Biol.

[17] Steinbacher T, Kummer D, Ebnet K (2018). Junctional adhesion molecule-A: functional diversity through molecular promiscuity. Cell Mol Life Sci CMLS.

[18] Iden S (2012). aPKC phosphorylates JAM-A at Ser285 to promote cell contact maturation and tight junction formation. J Cell Biol.

[19] Severson EA (2009). Junctional adhesion molecule A interacts with Afadin and PDZ-GEF2 to activate Rap1A, regulate beta1 integrin levels, and enhance cell migration. Mol Biol Cell.

[20] Naik MU (2008). Attenuation of junctional adhesion molecule-A is a contributing factor for breast cancer cell invasion. Cancer Res.

[21] Gotte M (2010). miR-145-dependent targeting of junctional adhesion molecule A and modulation of fascin expression are associated with reduced breast cancer cell motility and invasiveness. Oncogene.

[22] Murakami M (2011). Abrogation of junctional adhesion molecule-a expression induces cell apoptosis and reduces breast cancer progression. PLoS ONE.

[23] Kakuki T (2016). Dysregulation of junctional adhesion molecule-A via p63/GATA-3 in head and neck squamous cell carcinoma. Oncotarget.

[24] Naik MU, Vuppalanchi D, Naik UP (2003). Essential role of junctional adhesion molecule-1 in basic fibroblast growth factor-induced endothelial cell migration. Arterioscler Thromb Vasc Biol.

[25] Naik MU, Naik UP (2006). Junctional adhesion molecule-A-induced endothelial cell migration on vitronectin is integrin a_v_b_3_ specific. J Cell Sci.

[26] Peddibhotla SS (2013). Tetraspanin CD9 links junctional adhesion molecule-A to alphavbeta3 integrin to mediate basic fibroblast growth factor-specific angiogenic signaling. Mol Biol Cell.

[27] Rehder D (2006). Junctional adhesion molecule-A participates in the formation of apico-basal polarity through different domains. Exp Cell Res.

[28] Otani, T., et al., Claudins and JAM-A coordinately regulate tight junction formation and epithelial polarity. The Journal of cell biology, 2019. 10.1083/jcb.201812157 PMC6781433 31467165

[29] Fan S (2019). Role of JAM-A tyrosine phosphorylation in epithelial barrier dysfunction during intestinal inflammation. Mol Biol Cell.

[30] Ozaki H (2000). Junctional adhesion molecule (JAM) is phosphorylated by protein kinase C upon platelet activation. Biochem Biophys Res Commun.

[31] Merlen G (2019). TGR5-dependent hepatoprotection through the regulation of biliary epithelium barrier function. Gut.

[32] Poujade M (2007). Collective migration of an epithelial monolayer in response to a model wound. Proc Natl Acad Sci USA.

[33] Friedl P, Mayor R (2017). Tuning collective cell migration by cell-cell junction regulation. Cold Spring Harb Perspect Biol.

[34] Lopez-Colome AM (2017). Paxillin: a crossroad in pathological cell migration. J Hematol Oncol.

[35] McSherry EA (2009). JAM-A expression positively correlates with poor prognosis in breast cancer patients. Int J Cancer.

[36] McSherry EA (2011). Breast cancer cell migration is regulated through junctional adhesion molecule-A-mediated activation of Rap1 GTPase. Breast Cancer Res.

[37] Jokinen J (2004). Integrin-mediated cell adhesion to type I collagen fibrils. J Biol Chem.

[38] Humphries JD, Byron A, Humphries MJ (2006). Integrin ligands at a glance. J Cell Sci.

[39] Charrin S (2009). Lateral organization of membrane proteins: tetraspanins spin their web. Biochem J.

[40] Levy S, Shoham T (2005). Protein-protein interactions in the tetraspanin web. Physiology (Bethesda).

[41] Hemler ME (2005). Tetraspanin functions and associated microdomains. Nat Rev Mol Cell Biol.

[42] Yanez-Mo M (2009). Tetraspanin-enriched microdomains: a functional unit in cell plasma membranes. Trends Cell Biol.

[43] Naik MU (2003). Signaling through JAM-1 and {alpha}v{beta}3 is required for the angiogenic action of bFGF: dissociation of the JAM-1 and {alpha}v{beta}3 complex. Blood.

[44] Berditchevski F (2001). Complexes of tetraspanins with integrins: more than meets the eye. J Cell Sci.

[45] Yauch RL (2000). Direct extracellular contact between integrin alpha(3)beta(1) and TM4SF protein CD151. J Biol Chem.

[46] Yamada M (2008). Probing the interaction of tetraspanin CD151 with integrin alpha 3 beta 1 using a panel of monoclonal antibodies with distinct reactivities toward the CD151-integrin alpha 3 beta 1 complex. Biochem J.

[47] Scales TM (2013). alpha3beta1 integrins regulate CD151 complex assembly and membrane dynamics in carcinoma cells within 3D environments. Oncogene.

[48] Sterk LM (2002). Association of the tetraspanin CD151 with the laminin-binding integrins alpha3beta1, alpha6beta1, alpha6beta4 and alpha7beta1 in cells in culture and in vivo. J Cell Sci.

[49] Chattopadhyay N (2003). alpha3beta1 integrin-CD151, a component of the cadherin-catenin complex, regulates PTPmu expression and cell-cell adhesion. J Cell Biol.

[50] Baldwin G (2008). Tetraspanin CD151 regulates glycosylation of (alpha)3(beta)1 integrin. J Biol Chem.

[51] Gustafson-Wagner E, Stipp CS (2013). The CD9/CD81 tetraspanin complex and tetraspanin CD151 regulate alpha3beta1 integrin-dependent tumor cell behaviors by overlapping but distinct mechanisms. PLoS ONE.

[52] Novitskaya V (2014). Integrin alpha3beta1-CD151 complex regulates dimerization of ErbB2 via RhoA. Oncogene.

[53] Zevian SC (2015). CD151 promotes alpha3beta1 integrin-dependent organization of carcinoma cell junctions and restrains collective cell invasion. Cancer Biol Ther.

[54] Te Molder L (2019). Tetraspanin CD151 and integrin alpha3beta1 contribute to the stabilization of integrin alpha6beta4-containing cell-matrix adhesions. J Cell Sci.

[55] Yanez-Mo M (2001). Tetraspanins in intercellular adhesion of polarized epithelial cells: spatial and functional relationship to integrins and cadherins. J Cell Sci.

[56] Penas PF (2000). Tetraspanins are localized at motility-related structures and involved in normal human keratinocyte wound healing migration. J Invest Dermatol.

[57] Kawakami Y (2002). Tetraspanin CD9 is a "proteolipid," and its interaction with alpha 3 integrin in microdomain is promoted by GM3 ganglioside, leading to inhibition of laminin-5-dependent cell motility. J Biol Chem.

[58] Sobocka MB (2004). Signaling pathways of the F11 receptor (F11R; a.k.a. JAM-1, JAM-A) in human platelets: F11R dimerization, phosphorylation and complex formation with the integrin GPIIIa. J Recept Signal Transduct Res.

[59] Martin-Padura I (1998). Junctional adhesion molecule, a novel member of the immunoglobulin superfamily that distributes at intercellular junctions and modulates monocyte transmigration. J Cell Biol.

[60] Liu Y (2000). Human junction adhesion molecule regulates tight junction resealing in epithelia. J Cell Sci.

[61] Cera MR (2004). Increased DC trafficking to lymph nodes and contact hypersensitivity in junctional adhesion molecule-A-deficient mice. J Clin Investig.

[62] Bazzoni G (2005). Expression of junctional adhesion molecule-A prevents spontaneous and random motility. J Cell Sci.

[63] Farooqui R, Zhu S, Fenteany G (2006). Glycogen synthase kinase-3 acts upstream of ADP-ribosylation factor 6 and Rac1 to regulate epithelial cell migration. Exp Cell Res.

[64] Cera MR (2009). JAM-A promotes neutrophil chemotaxis by controlling integrin internalization and recycling. J Cell Sci.

[65] Haas AJ (2020). Interplay between extracellular matrix stiffness and JAM-A regulates mechanical load on ZO-1 and tight junction assembly. Cell Rep.

[66] Leal-Egana A (2017). The size-speed-force relationship governs migratory cell response to tumorigenic factors. Mol Biol Cell.

[67] Balasubramaniam L (2021). Investigating the nature of active forces in tissues reveals how contractile cells can form extensile monolayers. Nat Mater.

[68] Ladoux B, Mege RM (2017). Mechanobiology of collective cell behaviours. Nat Rev Mol Cell Biol.

[69] Jain S, Ladoux B, Mege RM (2021). Mechanical plasticity in collective cell migration. Curr Opin Cell Biol.

[70] Scott DW, Tolbert CE, Burridge K (2016). Tension on JAM-A activates RhoA via GEF-H1 and p115 RhoGEF. Mol Biol Cell.

[71] Ebnet K (2000). Junctional adhesion molecule interacts with the PDZ domain-containing proteins AF-6 and ZO-1. J Biol Chem.

[72] Spadaro D (2017). Tension-dependent stretching activates ZO-1 to control the junctional localization of its interactors. Curr Biol.

[73] Monteiro AC (2013). JAM-A associates with ZO-2, afadin, and PDZ-GEF1 to activate Rap2c and regulate epithelial barrier function. Mol Biol Cell.

[74] Winterwood NE (2006). A critical role for tetraspanin CD151 in alpha3beta1 and alpha6beta4 integrin-dependent tumor cell functions on laminin-5. Mol Biol Cell.

[75] Shigeta M (2003). CD151 regulates epithelial cell-cell adhesion through PKC- and Cdc42-dependent actin cytoskeletal reorganization. J Cell Biol.

[76] Ramovs V (2021). Integrin alpha3beta1 is a key regulator of several protumorigenic pathways during skin carcinogenesis. J Investig Dermatol.

[77] Naik MU, Caplan JL, Naik UP (2014). Junctional adhesion molecule-A suppresses platelet integrin alphaIIbbeta3 signaling by recruiting Csk to the integrin-c-Src complex. Blood.

[78] Horton ER (2016). Modulation of FAK and Src adhesion signaling occurs independently of adhesion complex composition. J Cell Biol.

[79] Tuncay H (2015). JAM-A regulates cortical dynein localization through Cdc42 to control planar spindle orientation during mitosis. Nat Commun.

[80] Meerbrey KL (2011). The pINDUCER lentiviral toolkit for inducible RNA interference in vitro and in vivo. Proc Natl Acad Sci USA.

[81] Taha AA, Hanbury A (2015). Metrics for evaluating 3D medical image segmentation: analysis, selection, and tool. BMC Med Imaging.

