# Supplementary material for: JAM-A interacts with α3β1 integrin and tetraspanins CD151 and CD9 to regulate collective cell migration of polarized epithelial cells
Source: Cell Mol Life Sci. 2022 Jan 24;79(2):88. doi: 10.1007/s00018-022-04140-5 (PMC8784505; doi:10.1007/s00018-022-04140-5)
Supplement: Supplementary file 1 — Supplementary file1 (PDF 764 KB) [file 18_2022_4140_MOESM1_ESM.pdf]

Supplementary Information

JAM-A interacts with α3β1 integrin and tetraspanins CD151 and CD9 to regulate collective cell migration of polarized epithelial cells

Cellular and Molecular Life Sciences

Sonja Thölmann, Jochen Seebach, Tetsuhisa Otani, Luise Florin, Hans Schnittler, Volker Gerke, Mikio Furuse, Klaus Ebnet*

*Author for correspondence
Klaus Ebnet, PhD, Institute-associated Research Group "Cell adhesion and cell polarity", Institute of Medical Biochemistry, ZMBE, University of Münster, Von-Esmarch-Str. 56, D-48149 Münster, Germany
e-mail: ebnetk@uni-muenster.de

Supplementary Figures S1 – S3

Supplementary Movie Legends

Supplementary Figures

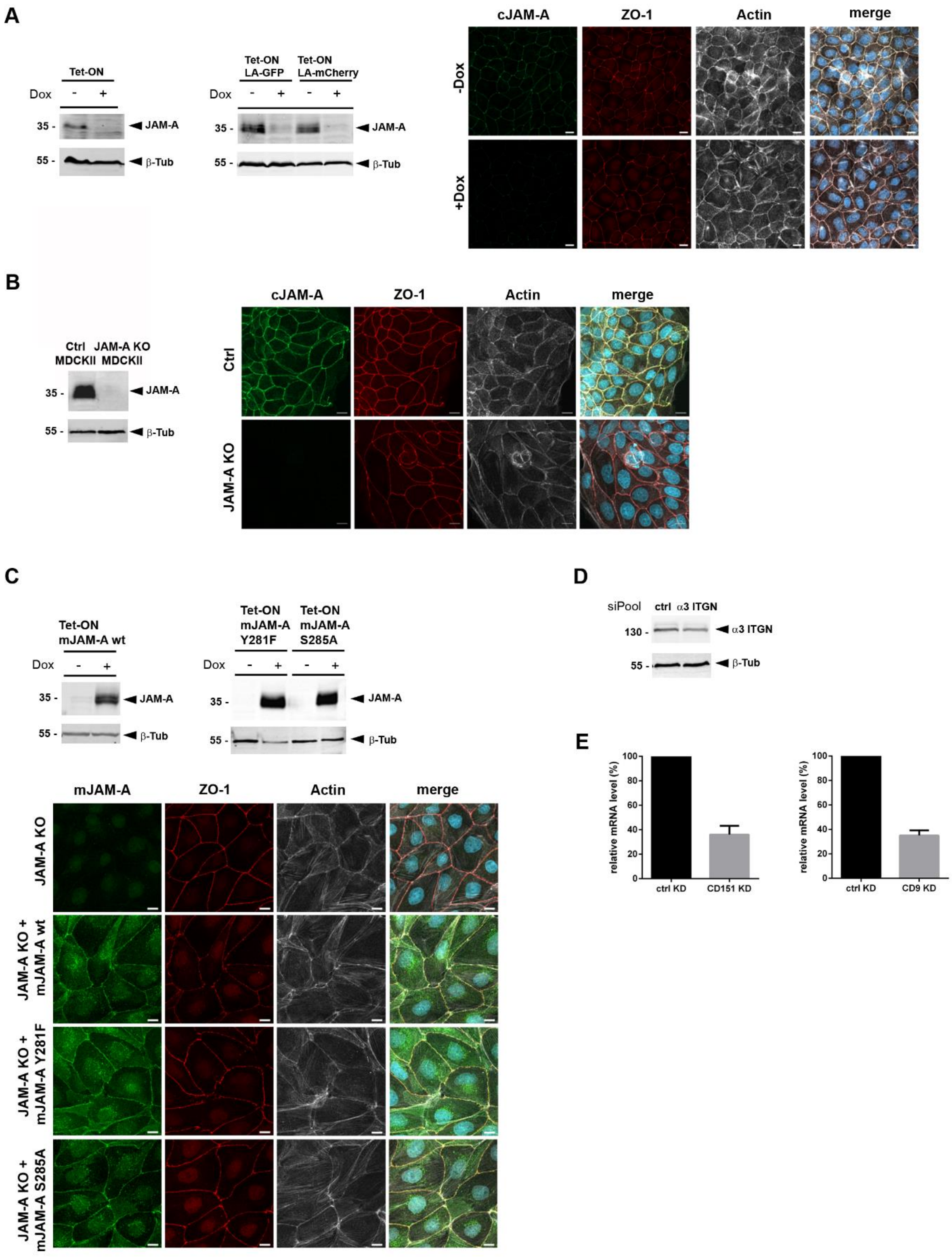

Suppl. Fig. S1: Analysis of knockdown efficiencies and characterization of JAM-A knockout MDCKII cells. (**A**) Knockdown efficiencies of JAM-A. Left: Western blot analysis of JAM-A in MDCKII

cells stably transfected with a conditional shRNA expression vector that expresses JAM-A shRNAs under a doxycycline-regulated promoter (pEmU6-proT, Tet-ON) without or with addition of doxycycline (-Dox or +Dox, respectively). Middle: Western blot analysis of JAM-A expression in pEmU6-proT/JAM-A shRNA-transfected MDCKII cells stably transfected with LA-EGFP or LA-mCherry without or with addition of doxycycline (-Dox or +Dox, respectively). Right: IF analysis JAM-A expression in pEmU6-proT/JAM-A shRNA-transfected MDCKII cells. Cells were stained for JAM-A, ZO-1 and F-Actin as indicated. Scale bars: 10 µm. (**B**) Characterization of JAM-A KO MDCKII cells. Left: Western blot analysis of JAM-A in MDCKII cells with a CrisprCas9-mediated inactivation of the JAM-A gene. Right: IF analysis of JAM-A KO MDCKII cells. Cells were stained for JAM-A, ZO-1 and F-Actin as indicated. Scale bars: 10 µm. (**C**) Characterization of JAM-A KO MDCKII cells reconstituted with expression vectors encoding murine JAM-A constructs (mJAM-A/WT, mJAM-A/Y281F, mJAM-A/S285A) under a doxycycline-regulated promoter (pInducer21, Tet-ON). Top: Western blot analysis of JAM-A constructs in the absence and presence of doxycycline (-Dox, +Dox, respectively). Bottom: IF analysis of JAM-A KO MDCKII cells after doxycycline-induced expression of murine JAM-A constructs. Cells were stained for mouse JAM-A, ZO-1 and F-Actin as indicated. Note that the ectopic JAM-A constructs localize to cell-cell contacts at comparable levels. Scale bars: 10 µm. (**D**) Knockdown efficiency of α3 integrin. Western blot analysis of the α3 integrin chain in LA-EGFP-expressing MDCKII cells after transient transfection with a control siRNA pool (ctrl) or a canine α3 integrin-specific siRNA pool (α3 ITGN). (**E**) Knockdown efficiencies of CD151 and CD9. Quantitative RT-PCR analysis of CD151 (left) and CD9 (right) in LA-EGFP-expressing MDCKII cells after transient transfection with a control siRNA pool (ctrl KD) or canine CD151-specific siRNA pool (CD151 KD) or canine CD9-specific siRNA pool (CD9 KD). Data are presented as mean values ± SD (n = 3 independent experiments).

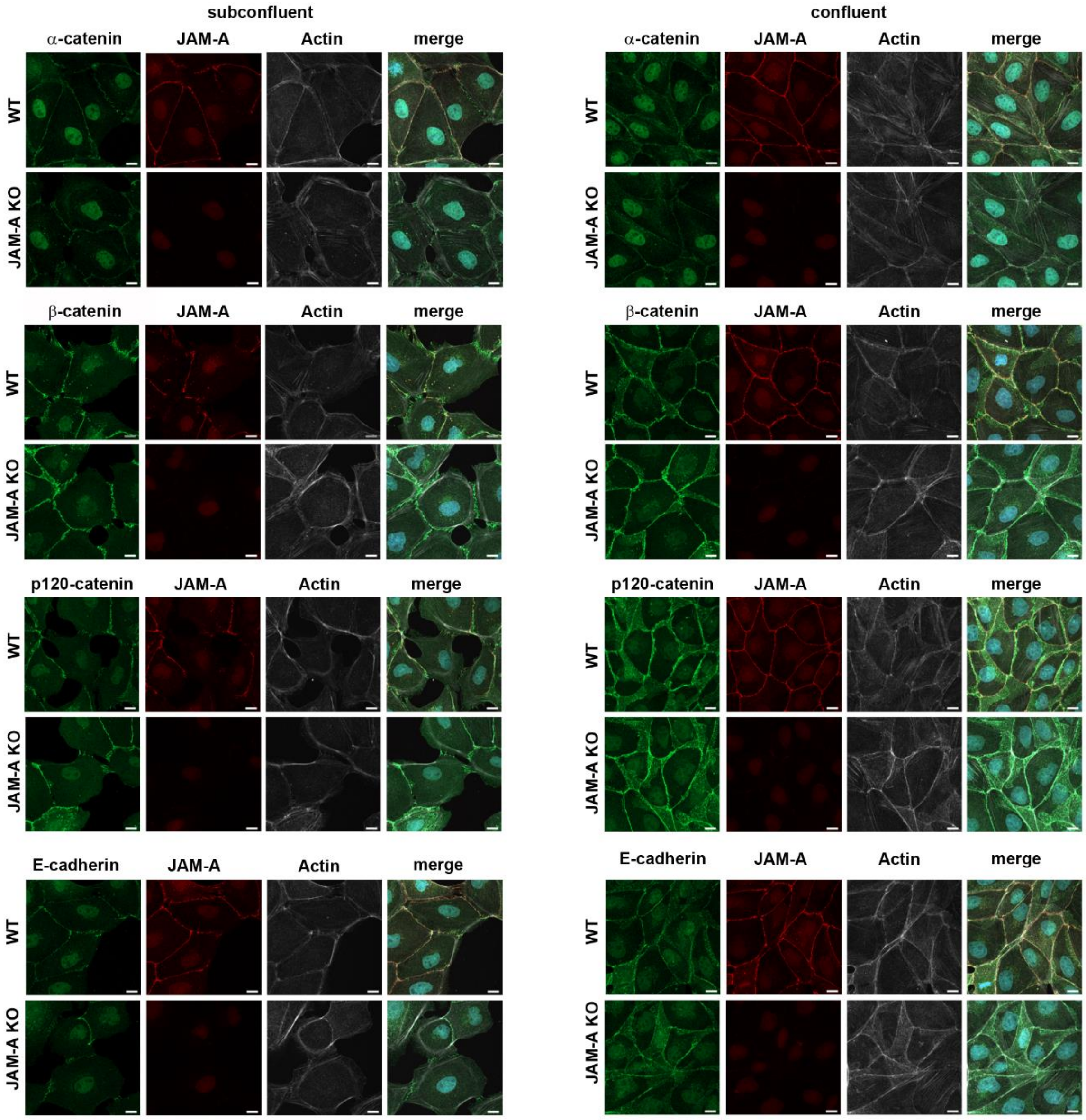

Suppl. Fig. S2: Characterization of adherens junctions proteins in JAM-A KO MDCKII cells reconstituted with mJAM-A constructs. MDCKII wildtype cells (WT) and JAM-A knockout MDCKII cells (JAM-A KO) were cultured at subconfluent conditions and confluent conditions, then fixed and stained with antibodies against α-catenin, β-catenin, p120-catenin, or E-cadherin as indicated. Note that all AJ proteins are normally localized at cell-cell contacts of JAM-A KO MDCKII cells. Scale bars: 10 µm.

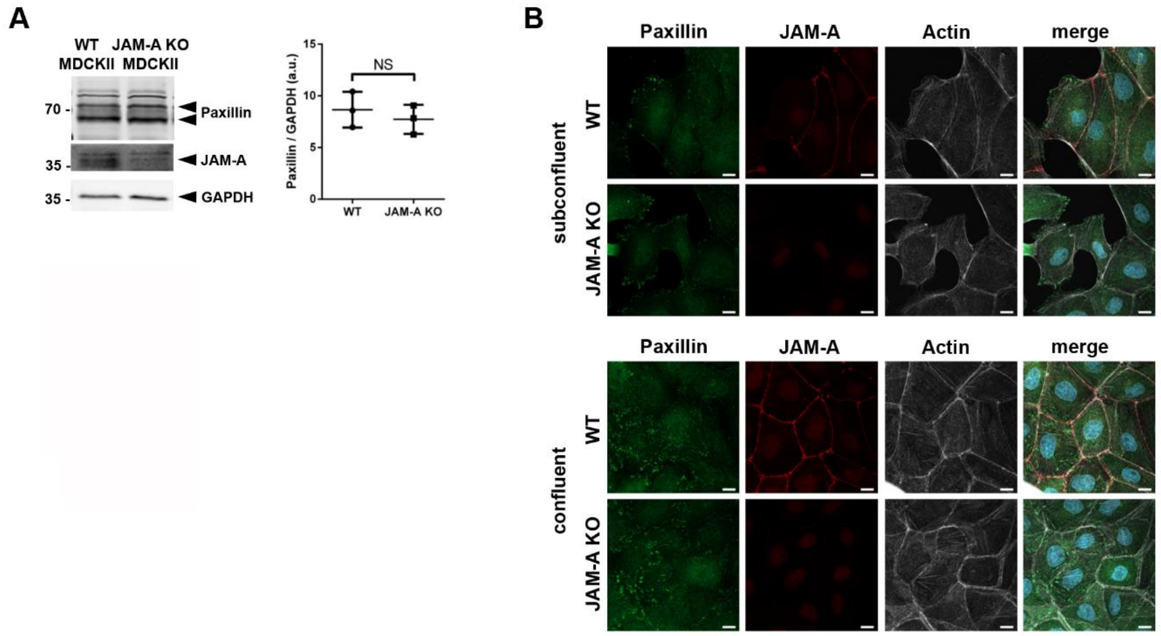

Suppl. Fig. S3: Characterization of paxillin expression and localization in JAM-A KO MDCKII cells. (**A**) Left: Western blot analysis of paxillin expression in JAM-A KO MDCKII cells. Right: Quantitative analysis of paxillin protein levels. Data are presented as mean values ± SD (n = 3 independent experiments). (**B**) IF analysis of paxillin-positive focal adhesions in JAM-A KO MDCKII cells cultured at subconfluent conditions and confluent conditions as indicated. Scale bars: 10 µm. Note that paxillin protein levels as well as paxillin-positive focal adhesions are unchanged in JAM-A KO cells.

Supplementary Movie Legends

Suppl. Movie 1 (SupplMovie1_single_cell_migration_ctrl.avi): **Single cell migration of a ctrl MDCKII cell**. Confocal laser scanning microscopy time-lapse movie of a LA-mCherry expressing MDCKII cell without induction of JAM-A shRNA expression (-Dox, ctrl). Z-stacks were taken every 10 minutes over a time-period of 10 h. Left: original video, right: analysis using the Trackmate plugin of ImageJ.

Suppl. Movie 2 (SupplMovie2_single_cell_migration_JAM-A_KD.avi): **Single cell migration of a JAM-A KD MDCKII cell**. Confocal laser scanning microscopy time-lapse movie of a LA-

EGFP expressing MDCKII cell with induction of JAM-A shRNA expression (+Dox, JAM-A KD). Z-stacks were taken every 10 minutes over a time-period of 10 h. Left: original video, right: analysis using the Trackmate plugin of ImageJ.

Suppl. Movie 3 (SupplMovie3_cryptic_lamellipodia_ctrl.avi): **Cryptic lamellipodia in ctrl MDCKII cells.** Confocal laser scanning microscopy time-lapse movie of mixed LA-EGFP and LA-mCherry expressing MDCKII cells without induction of JAM-A shRNA expression (-Dox, ctrl). Z-stacks were taken every 3 minutes over a time-period of 10 h. Left: original video with overlapping areas highlighted in yellow, right: analysis of overlapping areas by ImageJ.

Suppl. Movie 4 (SupplMovie4_cryptic_lamellipodia_JAM-A_KD.avi): **Cryptic lamellipodia in JAM-A KD MDCKII cells.** Confocal laser scanning microscopy time-lapse movie of mixed LA-EGFP and LA-mCherry expressing MDCKII cells with induction of JAM-A shRNA expression (+Dox, JAM-A KD). Z-stacks were taken every 3 minutes over a time-period of 10 h. Left: original video with overlapping areas highlighted in yellow, right: analysis of overlapping areas by ImageJ.

Suppl. Movie 5 (SupplMovie5_cryptic_lamellipodia_ctrl.avi): **Dynamic of cryptic lamellipodia in collectively migrating ctrl MDCKII cells.** Confocal laser scanning microscopy time-lapse movie of mixed LA-EGFP and LA-mCherry expressing MDCKII cells without induction of JAM-A shRNA expression (-Dox, ctrl). Z-stacks were taken every 3 minutes over a time-period of 10 h. Left: original video with overlapping areas highlighted in yellow, right: analysis of the dynamic of overlapping areas by ImageJ (increase in area shown in white, decrease in black).

Suppl. Movie 6 (SupplMovie6_cryptic_lamellipodia_JAM-A_KD.avi): **Dynamic of cryptic lamellipodia in collectively migrating JAM-A KD MDCKII cells.** Confocal laser scanning microscopy time-lapse movie of mixed LA-EGFP and LA-mCherry expressing MDCKII cells

with induction of JAM-A shRNA expression (+Dox, JAM-A KD). Z-stacks were taken every 3 minutes over a time-period of 10 h. Left: original video with overlapping areas highlighted in yellow, right: analysis of the dynamic of overlapping areas by ImageJ (increase in area shown in white, decrease in black).

Suppl. Movie 7 (SupplMovie7_single_cell_in_collective_ctrl.avi): **Migration behavior of single cells embedded in a cell collective (-Dox).** Confocal laser scanning microscopy time-lapse movie of mixed LA-EGFP and LA-mCherry expressing MDCKII cells without induction of JAM-A shRNA expression (-Dox, ctrl). Z-stacks were taken every 3 minutes over a time-period of 10 h. Left: original video, right: binary video of the single LA-mCherry expressing cell which were used to determine Jaccard indices, migration velocities and directionalities.

Suppl. Movie 8 (SupplMovie8_single_cell_in_collective_JAM-A_KD.avi): **Migration behavior of single cells embedded in a cell collective (+Dox).** Confocal laser scanning microscopy time-lapse movie of mixed LA-EGFP and LA-mCherry expressing MDCKII cells with induction of JAM-A shRNA expression (+Dox, JAM-A KD). Z-stacks were taken every 3 minutes over a time-period of 10 h. Left: original video, right: binary video of the single LA-mCherry expressing cell which were used to determine Jaccard indices, migration velocities and directionalities.
